# Advanced Heterostructured PEDOT–PB Transducer Interface via One-Step Progressive Electrochemical Deposition for Stable and High-Performance Hydrogen Peroxide Electrocatalysis

**DOI:** 10.1021/acsami.5c07083

**Published:** 2025-07-30

**Authors:** Kiattisak Promsuwan, Lingyin Meng, Panote Thavarungkul, Proespichaya Kanatharana, Warakorn Limbut, Wing Cheung Mak

**Affiliations:** † Division of Sensor and Actuator Systems, Department of Physics, Chemistry and Biology, 26686 Linköping University, Linköping SE-581 83, Sweden; ‡ Department of Biomedical Engineering, The Chinese University of Hong Kong, Hong Kong SAR, China; § Forensic Science Innovation and Service Center, Prince of Songkla University, Hat Yai, Songkhla 90110, Thailand; ∥ Center of Excellence for Trace Analysis and Biosensor, Prince of Songkla University, Hat Yai, Songkhla 90112, Thailand; ⊥ Division of Health and Applied Sciences, Faculty of Science, Prince of Songkla University, Hat Yai, Songkhla 90110, Thailand; # Division of Physical Science, Faculty of Science, Prince of Songkla University, Hat Yai, Songkhla 90110, Thailand

**Keywords:** prussian blue, poly(3, 4-ethylenedioxythiophene), electrochemistry, catalytic electrochemical interfaces, hydrogen peroxide reduction

## Abstract

Heterostructured organic–inorganic composites with syngenetic properties are highly appealing as advanced material interfaces for emerging electronic and bioelectronic device applications. For decades, Prussian blue (PB) has attracted tremendous attention due to its excellent and unique electrocatalytic properties as transducer materials, while conventional inorganic PB lacks sufficient stability and conductivity for advancing performance. Here, we demonstrate an innovative one-step progressive electrochemical deposition approach for the facile *in situ* fabrication of heterostructured Poly­(3,4-ethylenedioxythiophene) (PEDOT)–PB (*in situ* PEDOT–PB) transducer interface with excellent stability and electrocatalytic performance. The *in situ* PEDOT–PB was prepared by the simultaneous potentiodynamic oxidation of Fe­(II) precursors and electropolymerization of EDOT monomers, forming a porous and heterostructured PEDOT–PB interface with embedded PB nanoparticles. The *in situ* PEDOT–PB possessed 16.6- and 11.8-fold higher surface concentration (Γ) of PB, delivered excellent cycling stability (96.7% after 50 scanning cycles), enhanced electrode kinetics characterized by the lowest *R*_ct_ (0.57 Ω), and the highest catalytic rate constant (*K*_cat_) of 1238 M^–1^ s^–1^ toward hydrogen peroxide (H_2_O_2_) reduction compared with unary PB and stepwise PEDOT–PB. Moreover, the *in situ* PEDOT–PB showed good electrocatalytic stability for H_2_O_2_ reduction in a static system (3000 s) and a flow injection system (100 measurement cycles), retaining 80.7% and 96.6% of its initial catalytic performance, respectively. Our development provides a facile route for the design and preparation of high-performance, stable, and heterostructured PEDOT–PB for advanced electrochemical transducers for sensors and biosensors, electrocatalysis, and biofuel cell applications.

## Graphical abstract

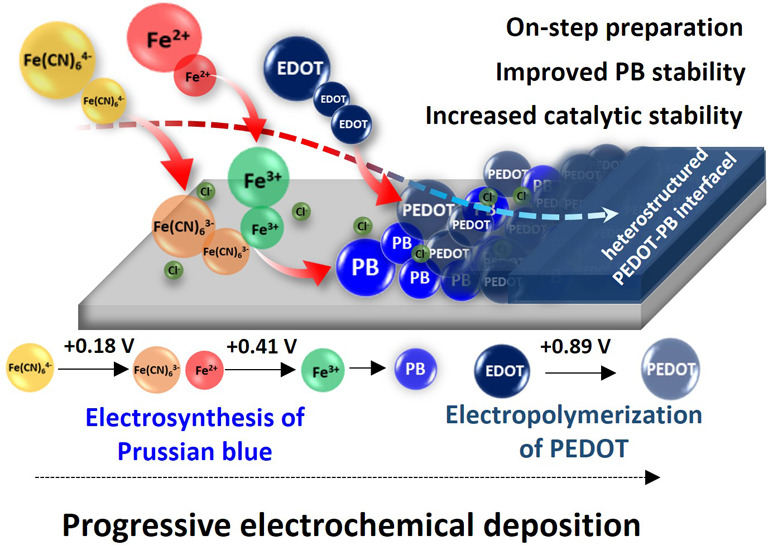

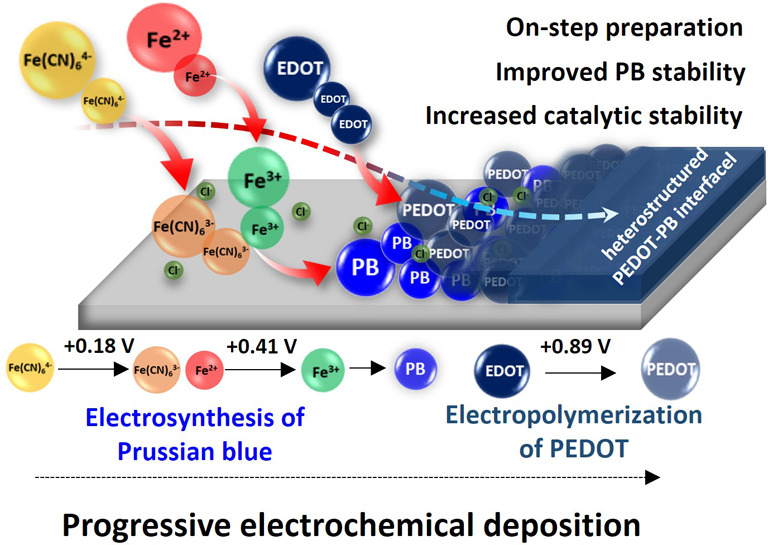

## 1 Introduction

Heterostructured organic–inorganic composites with syngenetic properties are highly appealing as advanced material interfaces for electrochemical applications. Prussian blue (PB), or ferric hexacyanoferrate, as the classical mixed-valence transition metal hexacyanometalate with peroxidase-like activity, has attracted tremendous attention due to its excellent electrochemical properties.^1^ Up to now, the synthesis and properties of PB nanoparticles have been well established and widely used as a transducing material for the construction of hydrogen peroxide sensors^2,3^ and oxidase-based electrochemical biosensors,^4,5^ owing to their excellent reversible redox properties and high electrocatalytic activity toward hydrogen peroxide reduction.^4^ However, PB has limited stability in practical applications since the local production of hydroxyl ions (OH^–^) and hydroxyl radicals (HO•) during hydrogen peroxide reduction could result in the dissolution of PB and release of soluble ferrocyanide and Fe^2+^.^6,7^ In contrast, bulk PB crystals could slow down the dissolution process; however, it is difficult to form dense films due to low film-forming ability and easy agglomeration. Furthermore, PB is a semiconductor with low electronic conductivity at room temperature, ranging from 10^–11^ to 10^–5^ S cm^–1^.^8^ Therefore, the development of novel preparation strategies for the PB nanostructure to achieve good stability, preserved electrocatalytic properties, and enhanced conductivity is essential for improved biosensing performance.

One strategy for improving PB stability and preventing agglomeration is by coating PB crystals via a hydrothermal method with various protective polymers such as polyvinylpyrrolidone (PVP),^9^ poly­(diallyldimethylammonium chloride) (PDDA),^10^ polyethylenimine (PEI),^46^ poly­(allylamine hydrochloride) (PAH),^11^ poly­(vinyl alcohol) (PVA),^11^ polystyrenesulfonate (PSS),^11^ polyethylene glycol (PEG),^12^ and poly­(D)-glucosamine (chitosan).^13^ However, this hydrothermal synthesis method does not realize *in situ* PB crystal formation on the electrode surface due to its low film-forming ability.^14^ Furthermore, coating PB with nonconductive polymers could affect the intrinsic PB electrocatalysis performance and electron transfer at the PB–electrode interface.^14^ Therefore, it is highly desirable to incorporate PB with a conductive matrix to improve operational stability and conductivity on electrode surfaces.

Electrochemical deposition is a versatile, efficient, and inexpensive technique for the synthesis of various nanostructured PB with conducting polymer coatings on a conductive substrate in the field of electrochemical sensors and biosensors.^15–17^ This technique features precise control over morphology, thickness, and prevention of agglomeration in the PB composition of the deposited coatings on the desired electrode surface, which can be tailored by electrical energy (e.g., potential), time, and precursor concentration during the nucleation and growth process.^15,18^ Beyond these advantages, the fabrication of heterogeneous PB composite materials via electrochemical deposition is of particular interest because of the synergistic properties of PB and conducting polymer components.

Poly­(3,4-ethylenedioxythiophene) (PEDOT) is one of the most promising organic conducting polymers, with the advantages of easy synthesis, good conductivity, and high stability, while exhibiting limited electrocatalytic activities.^19–21^ The integration of PB, which possesses excellent electrocatalytic properties, with PEDOT, known for its unique mixed ionic–electric conductivity, for the preparation of PEDOT–PB composites with synergistic catalytic activities, conductivity, and good stability, is desired. Conventional methods to prepare PEDOT–PB are mainly performed via stepwise approaches (i.e., multiple steps) and can be categorized into three types: 1) electropolymerization of PEDOT with Fe­(CN_6_)^3–^ as a dopant and subsequent conversion of Fe­(CN_6_)^3–^ into PB by potentiodynamic cycling in an Fe^3+^ solution;^22^ 2) electropolymerization of a PEDOT matrix on the electrode surface and successive electrodeposition of PB from Fe­(CN_6_)^3–^ and Fe^3+^ precursors;^23^ 3) chemical presynthesis of PB particles as a dispersant, followed by electropolymerization of PEDOT in the presence of presynthesized PB.^2,24^ These multistep approaches may not be effective for the integration of PB into PEDOT, are difficult to control, and may affect reproducibility and stability. Additionally, these procedures are time-consuming. Thus, the exploration of a one-step electrochemical deposition of the PEDOT–PB heterogeneous composite, in a manner of simultaneous polymerization of PEDOT and the *in situ* formation of PB nanoparticles, is attractive.

Hence, we introduced an innovative progressive electrochemical deposition method for the facile one-step preparation of a highly stable heterostructured PEDOT–PB electrode interface. During the progressive electrochemical deposition process, EDOT monomers were electropolymerized, forming a conducting PEDOT matrix, and simultaneously, the cycling electrochemical potential regulated the conversion of the Fe^2+^ and Fe­(CN_6_)^4–^ precursors, forming the PB nanoparticles, which were entrapped and protected by the conducting PEDOT matrix. The progressive electrochemistry not only provides a facile one-step approach for the preparation of heterostructured PEDOT–PB but also, more importantly, combines the electrochemical potential cycles to trigger the generation of the reactants (from the PB precursor) to control the PB nanoparticle formation and, at the same time, encapsulate the PB nanoparticles with polymerized conducting PEDOT, forming a heterostructured PEDOT–PB film with improved stability and performance. We studied and resolved the mechanism of the progressive electrochemical synthesis with cyclic and linear sweeping voltammetry techniques. The morphology and surface concentration (Γ) of PB of *in situ* heterostructured PEDOT–PB were evaluated. The electrochemical performance of *in situ* PEDOT–PB, including the electrode kinetics and catalytic stability toward the electrocatalytic reduction of H_2_O_2_ was investigated and compared with the unary PB and stepwise PEDOT–PB electrode interfaces.

## 2 Experimental Section

### 2.1 Materials

3,4-Ethylenedioxythiophene (EDOT) monomer (97%), ferrous chloride (FeCl_2_), ferric chloride (FeCl_3_), and potassium ferrocyanide (K_4_[Fe­(CN)_6_]) were purchased from Sigma-Aldrich. Potassium chloride (KCl) and hydrochloric acid (HCl) were purchased from Merck (Germany). 0.1 M phosphate buffer solution (PBS, pH 6.6) containing 0.1 M KCl was prepared by mixing K_2_HPO_4_ and KH_2_PO_4_ stock solution. All chemicals were of analytical grade and used without any further treatment. Deionized water (DI) with a resistivity not lower than 18.2 MΩ·cm, obtained from a purification system (Millipore Systems, USA), was used throughout.

### 2.2 Heterostructured PEDOT–PB Prepared by the *In Situ* Progressive Electrochemical Method

The progressive electrochemical preparation of heterostructured PEDOT–PB was performed by one-step potentiodynamic cycling of an electrode substrate in the EDOT monomer, Fe^2+^, and Fe­(CN_6_)^4–^ precursor solution. In brief, a 10 mM EDOT dispersion was prepared by dispersing 10.7 μL of EDOT monomer in 10 mL of HCl (0.01 M) under sonication for 30 min. The EDOT dispersion was then degassed by purging with nitrogen gas for 10 min, followed by the dissolution of K_4_[Fe­(CN)_6_] and FeCl_2_ with a concentration of 5 mM, respectively. A glassy carbon electrode (GCE, diameter 3 mm) was polished with 0.3 and 0.05 μm aluminum slurries. The polished GCE was rinsed with water and sonicated in deionized water and ethanol for 2 min, respectively, and then dried under nitrogen gas. Heterostructured PEDOT–PB was deposited on the GCE by potentiodynamic cycling for 10 cycles at a scan rate of 0.05 V s^–1^ in the range from −0.50 to 1.20 V. The potential window of −0.50 to 1.20 V vs Ag/AgCl was selected to enable the simultaneous electrodeposition of PEDOT and Prussian Blue (PB) while avoiding degradation of either material. The anodic limit at 1.20 V corresponds to the onset oxidation potential of EDOT, which promotes efficient PEDOT polymerization with high conductivity and stability.^25^ The cathodic limit at −0.50 V was chosen to minimize the over-reduction of PEDOT, which can lead to conductivity loss and undesired oxygen reduction side reactions.^26^ This window also encompasses the deposition range for PB, typically occurring around 0.20 to 0.40 V,^6^ ^6^ enabling hybrid film formation under a single-step CV process.

After that, the electrode interface was cleaned to remove the excess solution with deionized water and was denoted as *in situ* PEDOT–PB. In comparison, the PEDOT film was prepared via the electropolymerization of EDOT monomers. PB was electrochemically deposited on the electrode surface by CV using K_4_[Fe­(CN)_6_] and FeCl_2_ as the precursors. For the stepwise integration of PB within the PEDOT matrix, PEDOT was electropolymerized in EDOT monomer using K_4_[Fe­(CN)_6_] as the dopant, followed by the potentiodynamic cycling in FeCl_2_ for the formation of PB, which was denoted as stepwise PEDOT–PB. In addition, chemically synthesized PB integrated with the PEDOT film was prepared by electropolymerization of EDOT monomer in the presence of chemically synthesized PB NPs, which was also denoted as stepwise PEDOT–PB_chemical_. Detailed information on the influence of scan rate (Figure S1A and Table S1) and deposition cycles (Figure S1B and Table S2) on *in situ* PEDOT–PB film formation, as well as procedures for the preparation of the comparison samples with their corresponding CVs (Figure S2) are provided in the Supporting Information.

### 2.3 Surface Characterizations and Electrochemical Measurements

Scanning electron microscopy (SEM, LEO 155 Gemini, Zeiss, Germany) was used to record images of the surface morphologies of the PB, PEDOT, and PEDOT–PB interfaces. Energy-dispersive X-ray spectroscopy (EDS, Oxford Instruments) was employed to determine the chemical compositions. Fourier transform infrared (FTIR) spectrometric measurements were performed by a VERTEX (Bruker), equipped with an attenuated total reflection (ATR) measuring cell, for investigating the characteristic bands. All electrochemical experiments were carried out by a conventional three-electrode system comprising a platinum wire as the counter electrode, a glassy carbon working electrode, and a silver/silver chloride (Ag/AgCl) electrode as the reference. Cyclic voltammetry (CV) with a scan rate of 0.05 V s^–1^ was performed for the electrodeposition, electrochemical behavior in PBS, and cycle stability evaluation of the modified electrodes. LSV measurements were performed in the potential range from −0.50 to 1.20 V at a scan rate of 0.01 V s^–1^ to prove the mechanism of simultaneous electrodeposition of the *in situ* PEDOT–PB composite. Electrochemical impedance spectroscopy (EIS) was applied for the electrochemical characterization of each modified electrode in 0.1 M KCl containing 5 mM [Fe­(CN)_6_]^3–/4–^ solution in the frequency range of 100 kHz–0.1 Hz. All electrochemical measurements were performed with a PalmSens4 (PalmSens BV, Netherlands) controlled by PSTrace 5.9 software at room temperature.

## 3 Results and Discussions

### 3.1 Mechanism of the Progressive Electrochemical Synthesis

*In situ* PEDOT–PB was progressively deposited on the electrode surface via one-step potentiodynamic cycling with EDOT and Fe^2+^/Fe­(CN_6_)^4–^ as the precursors for PEDOT and PB, respectively, and the corresponding cyclic voltammograms (CVs) are shown in Figure 1A. As shown in Figure 1A, the capacitance and redox peak currents gradually increased with the increasing number of cycles, indicating the successful growth of PEDOT and PB on the electrode surface.^27–29^ When focusing on the first cycle of CV (Figure 1B), a couple of redox peaks in the potential range from 0.00 to 0.60 V, and an oxidation process over the range of 0.90 to 1.20 V were observed, which are ascribed to the PB formation/redox reaction^29^ and the oxidation/polymerization process of EDOT,^27^ respectively. The progressive electrodeposition of *in situ* PEDOT–PB follows: 1) during the positive scan up to 0.6 V, the precursor Fe^2+^ and Fe­(CN)_6_^4–^ are oxidized to Fe^3+^ and Fe­(CN)_6_^3–^, respectively, at the electrode surface; 2) the new formation of Fe (III) species react with the existing Fe (II) species forming PB nanoparticles;^30^ 3) successive positive scans result in the oxidation of the EDOT monomer and its polymerization into PEDOT, while *in situ* embedding the PB nanoparticles in the PEDOT matrix.

**Figure 1:**
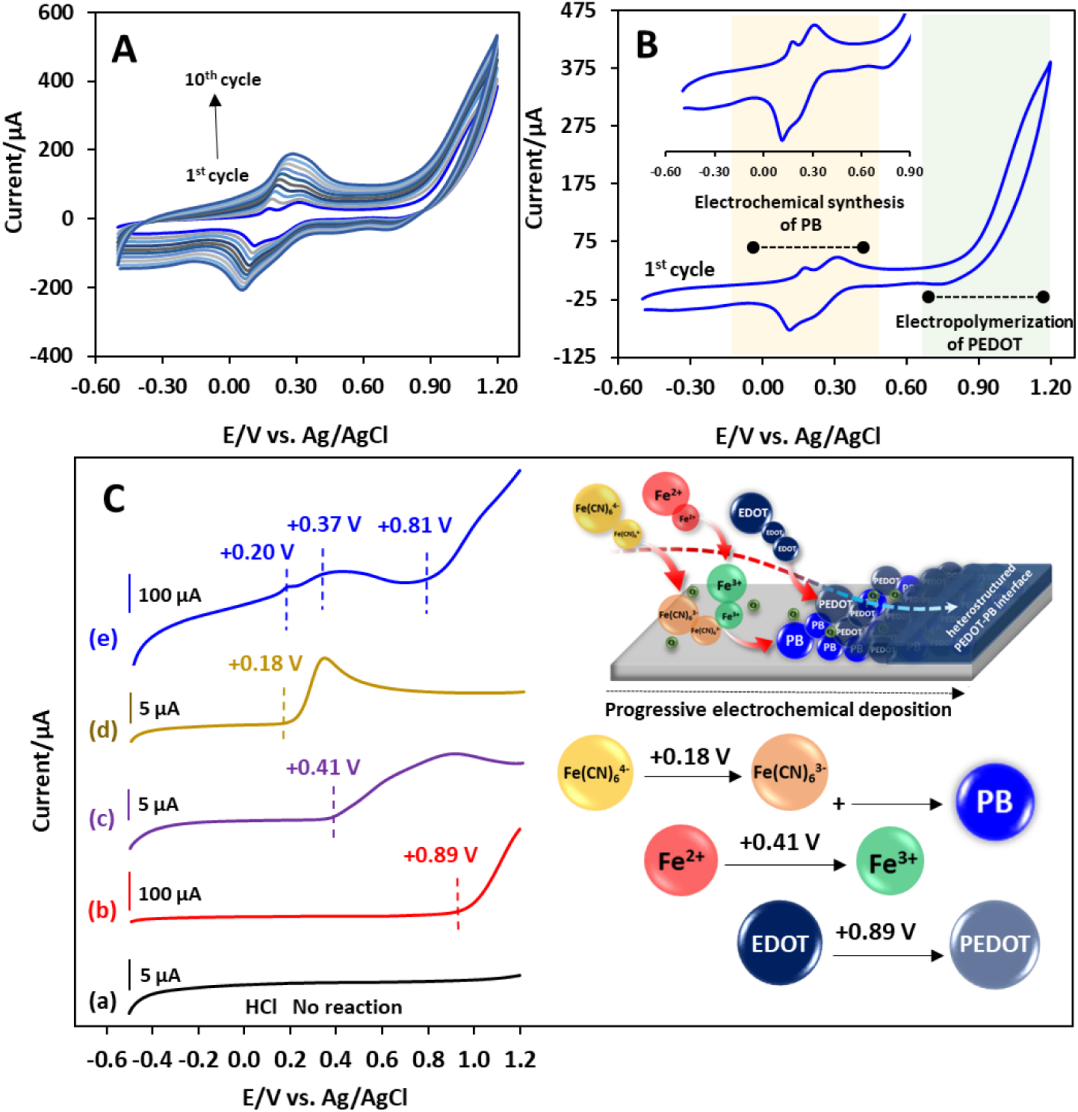
(A) 10 cycles and (B) the 1st cycle of CVs for the progressive electrochemical synthesis of *in situ* PEDOT–PB on a GCE in 10 mM HCl containing 10 mM EDOT, 5 mM FeCl_2_, and 5 mM K_4_Fe­(CN_6_) at a scan rate of 0.05 V s^–1^. (C) LSVs of GCE in (a) 10 mM HCl and 10 mM HCl containing (b) 10 mM EDOT, (c) 5 mM FeCl_2_, (d) 5 mM K_4_Fe­(CN_6_), and (e) a mixture of 5 mM K_4_Fe­(CN_6_), 5 mM FeCl_2_, and 10 mM EDOT at a scan rate of 0.01 V s^–1^.

To prove the mechanism of simultaneous electrodeposition of PEDOT–PB, the LSV measurement was performed in different precursor solutions. As shown in Figure 1C, no oxidation peaks were noted in the HCl solution during the positive scan (curve a). For the solution containing EDOT monomers, the LSV curve (curve b) showed an oxidation process with an onset potential of 0.89 V, which implies the formation of radical cations before the nucleation growth of PEDOT on the electrode surface.^27,28^ A broad oxidation peak over the range of 0.41 to 1.20 V can be observed for FeCl_2_ (curve c), which was the result of Fe^2+^ changing to Fe^3+^.^31,32^ The LSV curve for K_4_Fe­(CN_6_) (curve d) displayed a distinct oxidation peak with an onset potential of 0.18 V due to the oxidation of Fe­(CN)_6_^4–^ species to Fe­(CN)_6_.^3–17,32^ For the LSV curve from the mixture of EDOT, FeCl_2_, and K_4_Fe­(CN_6_) precursors (curve e), there are several characteristic peaks for the oxidation of Fe­(II) species and EDOT monomers that are consistent with curves a–d. It should be noted that the weak shoulder peak at around 0.20 V might be contributed by the oxidation of either Berlin white Fe_2_[Fe­(CN)_6_], formed by the complex of two Fe­(II) species (i.e., Fe^2+^ and Fe­(CN)_6_^4–^), or a small amount of PB formed by the air-oxidized Fe^3+^ from Fe^2+^ and its reaction with Fe­(CN)_6_.^2–4,22,33^ Beyond this, the oxidation peak of Fe­(CN)_6_^4–^ overlaid with the oxidation peak of Fe^2+^, resulting in the broadness of the oxidation peak at around 0.37 V. The onset potential for the oxidation/polymerization of EDOT into PEDOT decreased to around 0.81 V, which might be caused by the assistant doping effect from Fe­(CN)_6_^4–^. Furthermore, the magnification of the current for the progressive electrodeposition of *in situ* PEDOT–PB is much higher than that of the LSV curve from individual precursor solutions, indicating an improved synergistic efficiency for the electrochemical deposition. The interaction mechanism between PB and PEDOT can be described as a combination of electrostatic and chemical interactions. PEDOT, in its doped form, carries a positively charged π-conjugated backbone, while PB typically exhibits a negatively charged surface under neutral to mildly acidic conditions.^22,34^ This contrast in surface charge facilitates electrostatic attraction, promoting stable incorporation of PB within the PEDOT matrix. In addition to these electrostatic effects, coordination bonding between the two components is also likely. Specifically, iron atoms from PB may interact with sulfur atoms in PEDOT through electron pair donation, leading to the formation of Fe–S coordination bonds,^23^ which is confirmed by XPS results (Figure S3). These synergistic interactions contribute to the structural integrity, enhanced electron transfer, and improved electrochemical performance of the PEDOT–PB composite, making it a promising material for sensing, electrocatalytic and energy-related applications.

### 3.2 Surface Characterizations

Surface morphologies of the electrochemically deposited PEDOT, unary PB, stepwise PEDOT–PB, and *in situ* PEDOT–PB electrode interfaces are shown in Figure 2A–D. The PEDOT interface shows a compact film with a coarse, spongy-like morphology resulting from the Cl^–^ as counterion electrolyte^35^ (Figure 2A). The unary PB electrode is covered with a layer of PB nanoparticles (Figure 2B). For the stepwise PEDOT–PB interface, it consists of a compact base layer of PEDOT with cauliflower-like structures resulting from the Fe­(CN)_6_^4–^ counterion electrolyte,^22,36^ followed by an upper layer of larger size PB nanoparticles (Figure 2C). The relatively larger size of the PB nanoparticles can be explained by the stepwise deposition process. During the second step, the electrochemically converted Fe^3+^ reacts with the preloaded precursor Fe­(CN)_6_^4–^ within the PEDOT layer to support the nucleation and growth of larger-sized PB nanoparticles drafted on the surface of the PEDOT layer.^22,37^ The *in situ* PEDOT–PB interface shows a porous and heterostructured PEDOT film embedded with PB nanoparticles (Figure 2D). The porous structure can be explained by the *in situ* formation of PB nanoparticles, which hinder the agglomeration of PEDOT during the polymerization process. The macroscopic appearance of the *in situ* PEDOT films displays a dark blue to nearly black color with moderate reflectivity compared to PB and PEDOT films (Figure S4), indicating increased density and complexity at the surface. The EDS spectrum (Figures 2E and S5) and mapping (Figure S6) of the *in situ* PEDOT–PB nanocomposite display the existence of signals for the O, S, C, Fe, N, Cl, and K elements, which are from the conductive matrix of PEDOT (O, S, C, and Cl elements) and the PB catalyst (Fe, N, and K elements). In addition, as shown in the secondary SEM images and corresponding EDS maps, PEDOT (Figure S6A) exhibits a relatively rough and textured surface with a strong sulfur (S) signal, consistent with a continuous polymeric matrix. PB (Figure S6B) presents a homogeneous distribution of iron (Fe), characteristic of the PB framework. The stepwise PEDOT–PB composite (Figure S6C) shows a relatively distinguishable, well-defined PB-rich domain (indicated by localized Fe signals) distributed on or within the PEDOT matrix (indicated by S signals). These PB domains exhibit a cubic morphology with lateral dimensions ranging from approximately 200 to 500 nm. In contrast, the *in situ* PEDOT–PB composite (Figure S6D) displays a surface with richer PB domains (Fe content) embedded in the PEDOT background. In this case, the PB particle size is not clearly distinguishable, as the PEDOT matrix appears to fill the interparticle spaces, contributing to a less defined microstructure. The percentage data of the elemental atoms for quantitative comparison of each electrode interface are provided in Table S3. Considering the S/C and Fe/C ratios, the *in situ* PEDOT–PB interface exhibits higher values compared to the stepwise PEDOT–PB configuration. This suggests a more effective incorporation of both sulfur-containing PEDOT and iron-containing PB components, which may enhance the interfacial conductivity and redox activity, thereby improving the overall electrocatalytic performance.

**Figure 2:**
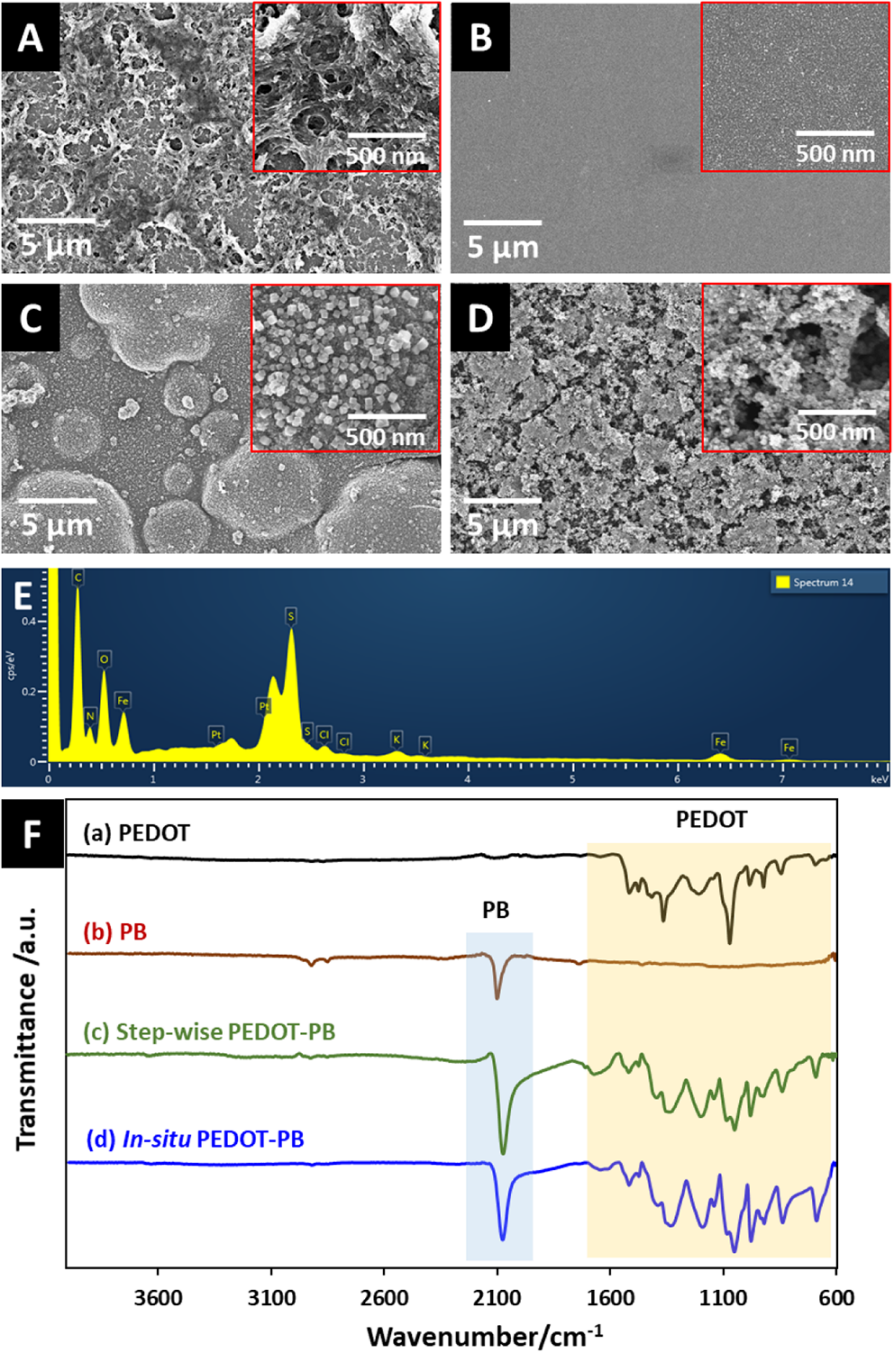
SEM images of (A) PEDOT, (B) PB, (C) stepwise PEDOT–PB, and (D) *in situ* PEDOT–PB. (E) EDS spectrum of *in situ* PEDOT–PB. (F) FTIR spectra of (a) PEDOT, (b) PB, (c) stepwise PEDOT–PB, and (d) *in situ* PEDOT–PB.

FTIR measurement was carried out to reveal the chemical characteristics of each modified electrode. Figure 2E shows the FTIR spectra of PEDOT, unary PB, stepwise PEDOT–PB, and *in situ* PEDOT–PB, respectively. As shown in Figure 2F­(a), the FTIR spectrum of PEDOT shows the main characteristic bands at 1510, 1310, and 1155 cm^–1^, which are attributed to the asymmetric stretching mode of C=C, C–C, and the stretching mode of C–O–C, respectively.^19,38^ The C–S bond stretching vibrations in the thiophene ring can be observed at 762 cm^–1^.^20,38,39^ For the FTIR spectrum of unary PB, Figure 2F­(b) shows a sharp peak at 2098 cm^–1^ corresponding to the cyanide stretching vibration mode (C≡N) in the Fe­(II)-C≡N–Fe­(III) bridge.^40^ Both the stepwise PEDOT–PB in Figure 2F­(c) and *in situ* PEDOT–PB in Figure 2F­(d) display the characteristic bands originating from PEDOT and PB, with a small drifting of the absorption band of PB due to the interaction of PEDOT with the inorganic PB. Additionally, the SEM image of the *in sit*u PEDOT–PB also showed more porosity than the chemically synthesized PB integrated with PEDOT, which is a relatively compact film of PEDOT with PB nanoparticles entrapped inside, and its FTIR spectrum and EDX results showed the same as *in situ* PEDOT–PB (details in Figure S7). Moreover, to assess the mechanical adhesion of the *in situ* PEDOT–PB coating, a sonication test was performed under deliberately harsh conditions. Although sonication is not applied during normal electrochemical operation, the coating maintained excellent structural integrity without noticeable morphological changes or peeling after 2 min of continuous ultrasonic agitation (Figure S8), indicating strong mechanical adhesion and robustness of the film.

### 3.3 Electrochemical Characterizations

To characterize the electrochemical interface properties of different modified electrodes, CV and EIS were applied. Figure 3A shows the CV curves of bare GCE (a), PB (b), PEDOT (c), stepwise PEDOT–PB (d), and *in situ* PEDOT–PB (e) in 0.1 M PBS at a scan rate of 0.05 V s^–1^. The *in situ* PEDOT–PB exhibited the largest redox peak currents and capacitive current when compared to the bare GCE, unary PB, PEDOT, and stepwise PEDOT–PB. The redox peak currents originate from the redox reaction of Fe­(II)/Fe­(III) species in PB. The anodic peak current of the *in situ* PEDOT–PB is 216.2 μA, which is 18.5 and 8.4 times higher than that of PB (11.1 μA) and stepwise PEDOT–PB (23.1 μA), respectively. Additionally, the surface concentration (Γ) of PB on the electrode surfaces was investigated by calculation using the reduction peak according to the following eq 1:^41^

1 Γ=Q/nFA

where *Q* is the charge value estimated by integrating the reduction peak (Table S4), *n* is the number of electrons in the reaction, *F* is Faraday’s constant, and *A* is the geometric area of the electrode. The values of Γ were found to be 1.59, 2.23, and 26.43 nmol cm^–2^ for PB, stepwise PEDOT–PB, and *in situ* PEDOT–PB, respectively. The amount of PB for one-step *in situ* PEDOT–PB is about 16.6- and 11.8-fold higher than that of unary PB and stepwise PEDOT–PB, respectively, which is in good agreement with the trend in anodic peak currents obtained from CVs shown in Figure 3A (i.e., the electrode with a higher amount of PB shows a larger anodic peak current). Moreover, the high PB loading amount of the one-step *in situ* PEDOT–PB is consistent with the morphological characteristics of the *in situ* PEDOT–PB, with a porous heterostructured PEDOT film embedded with PB nanoparticles (Figure 2D).

**Figure 3:**
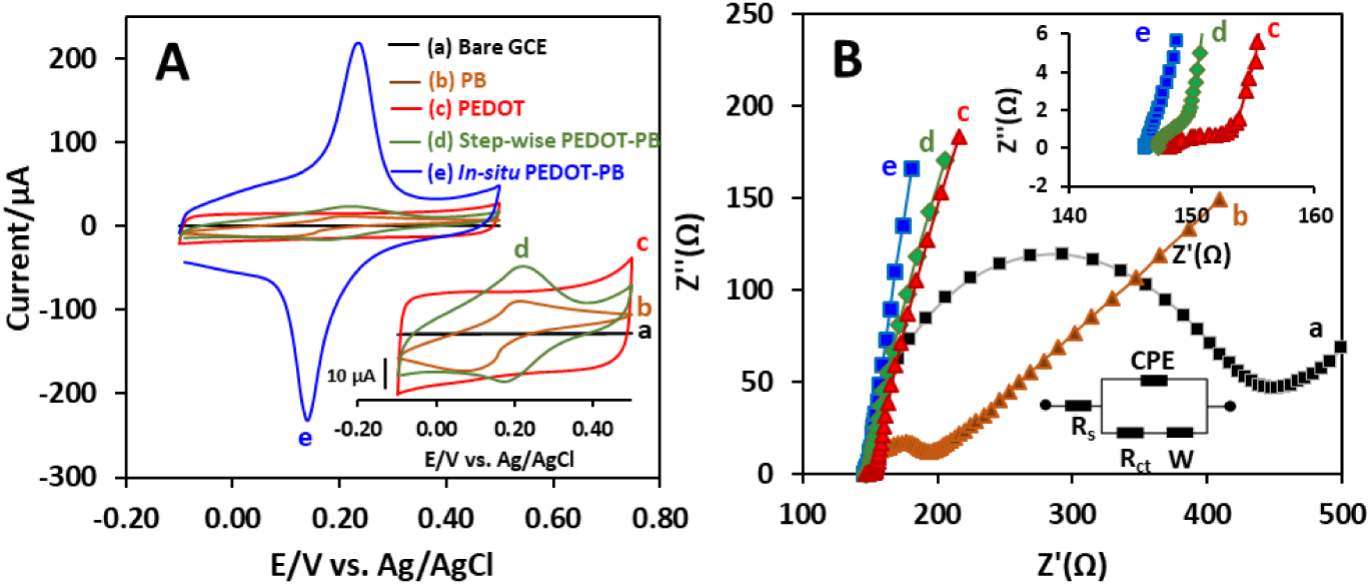
(A) CVs of bare GCE (a), PB (b), PEDOT (c), stepwise PEDOT–PB (d), and *in situ* PEDOT–PB (e) electrodes in PBS at a scan rate of 0.05 V s^–1^. (B) Nyquist plots for EIS measurements of bare GCE (a), PB (b), PEDOT (c), stepwise PEDOT–PB (d), and *in situ* PEDOT–PB electrodes in 0.1 mM KCl containing 5 mM Fe­(CN_6_)^3–/4–^ at a frequency range of 0.1–100 kHz.

Figure 3B displays the Nyquist plots of bare GCE (a), PB (b), PEDOT (c), stepwise PEDOT–PB (d), and *in situ* PEDOT–PB (e) from EIS measurements in 0.1 M KCl containing 5 mM Fe­(CN_6_)^3–/4–^ solution over the frequency range of 100 kHz–0.1 Hz. As shown in Figure 3B, all EIS spectra exhibited a semicircle in the high-frequency region corresponding to the charge transfer process, in which the semicircle diameter value represents the charge transfer resistance (*R*_ct_). Besides, a linear part at low frequency is related to the diffusion process at the electrode interface. The EIS results were fitted by the equivalent Randles circuit (inset of Figure 3B), and the corresponding fitting parameters are listed in Table S5. The results show that the *in situ* PEDOT–PB has the lowest R_ct_ value of 0.57 Ω compared to the bare GCE (297.06 Ω), unary PB (71.03 Ω), PEDOT (17.01 Ω), and stepwise PEDOT–PB (4.72 Ω), respectively. The improved impedance property of the *in situ* PEDOT–PB could be attributed to the fast electron transfer enabled by the synergistic effect of PB in the conductive PEDOT matrix.^24^ Additionally, the charge transfer rate constant (*K_s_*) of each modified electrode was calculated from *R*_ct_ using eq 2.^42,43^

2 $$R_{\mathrm{ct}}=\mathrm{RT}/n^{2}F^{2}K_{s}C^{\prime }$$

where *n*, *T*, *C*’, *R*, and *F* are the number of electrons of the redox probe, temperature, concentration of the redox probe, the gas constant, and Faraday’s constant, respectively. The *K_s_* values were found to be 1.8 × 10^–7^, 7.5 × 10^–7^, 3.1 × 10^–6^, 1.1 × 10^–5^, and 9.3 × 10^–5^ cm s^–1^ for the bare GCE, unary PB, PEDOT, stepwise PEDOT–PB, and *in situ* PEDOT–PB, respectively. The higher *K_s_* value of the *in situ* PEDOT–PB confirms better charge transfer efficiency within the heterostructured PEDOT–PB composite. To complement the Nyquist plots, Bode plots were added to provide explicit frequency-dependent information, offering a more comprehensive understanding of the impedance behavior of each modified electrode (Figure S9).

The stability of PB at the unary PB, stepwise PEDOT–PB, and *in situ* PEDOT–PB modified electrodes was evaluated via CV scanning between −0.10 and 0.50 V for 50 cycles. As shown in Figure 4A, the CVs of the unary PB electrode show a dramatic decrease in the redox peak currents with the increase in cycling number, which is caused by the decomposition of unary PB by hydroxide ions or the leakage of PB nanoparticles from the electrode surface. For the stepwise PEDOT–PB (Figure 4B), an apparent decay of the redox current can still be observed despite some improvement in stability by the PEDOT matrix. This is likely due to the formation of PB on the PEDOT surface rather than within the matrix during the stepwise electrochemical deposition. In comparison, the *in situ* PEDOT–PB electrode (Figure 4C) shows good stability without an apparent decrease in the current. Figure 4D illustrates the anodic peak current change during cycling with the first cycle set as 100%. The *in situ* PEDOT–PB electrode retains 97% of its initial value after 50 cycles, which is much higher than that of PB (15%) and stepwise PEDOT–PB (51%). In addition, the *in situ* PEDOT–PB also possesses better cycling stability compared to the stepwise PEDOT–PB_chemical_ (83%) (details in Figure S10A). This improvement in stability can be attributed to the *in situ* embedding and simultaneous fabrication of PB nanoparticles in the PEDOT matrix during the progressive electrochemical deposition process. Due to the limited stability of Prussian Blue (PB), the long-term stability of PB and its composites has not been extensively addressed in the previously reported literature. Therefore, to provide new insights into the long-term performance of *in situ* PEDOT for practical applications, an extended cycling test was conducted by subjecting the *in situ* PEDOT–PB transducer to 1,000 consecutive potential cycles. The results revealed that the current response retained approximately 72% of its initial value after 1,000 cycles, indicating a moderate decline in performance under prolonged operation (Figure S11A,B), demonstrating the potential for practical applications.

**Figure 4:**
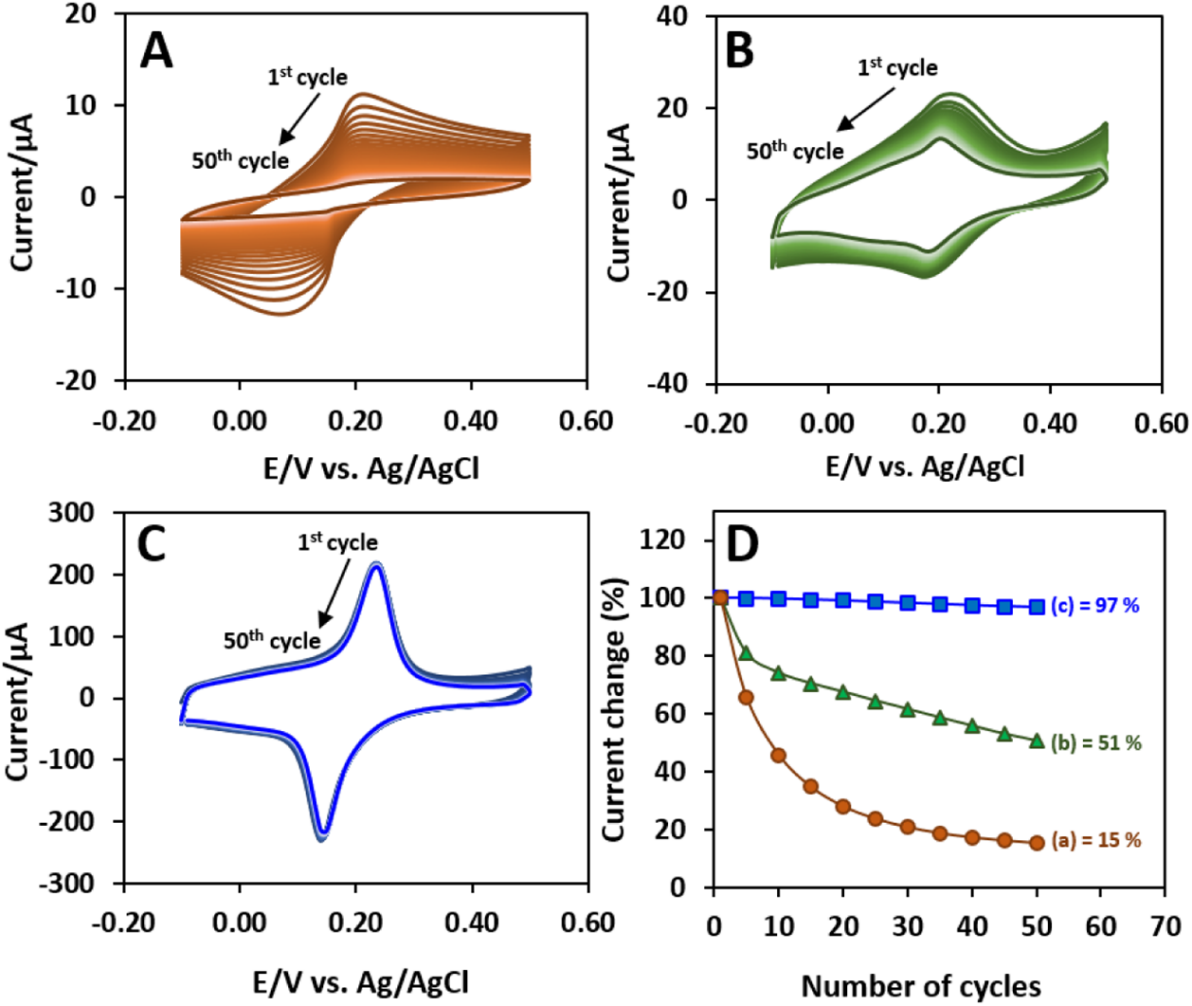
CVs of (A) PB, (B) stepwise PEDOT–PB, and (C) *in situ* PEDOT–PB in PBS at a scan rate of 0.05 V s^–1^. (D) Anodic peak current changes of (a) PB, (b) stepwise PEDOT–PB, and (c) *in situ* PEDOT–PB over different numbers of cycles.

### 3.4 Electrochemical Catalytic Performance for H_2_O_2_ Reduction

The electrochemical catalytic reaction toward H_2_O_2_ is important in various fields, such as sensors, enzyme-relayed biosensors, biofuel cells, electrocatalysis, and energy conversion technologies.^33,44^ Therefore, H_2_O_2_ was selected as the model substrate to evaluate the electrocatalytic ability of the prepared PEDOT–PB interface. The electrochemical catalytic performance of the modified electrode for H_2_O_2_ was investigated by CV and chronoamperometry. Figure 5A–C displays the CV curves of the unary PB, stepwise PEDOT–PB, and *in situ* PEDOT–PB in the absence and presence of 4 mM H_2_O_2_ at a scan rate of 50 mV s^–1^. In the absence of H_2_O_2_, all of the electrodes showed the characteristic redox peak associated with PB. After the addition of H_2_O_2_, all of the electrodes displayed the reduction peaks associated with PB reduction in response to H_2_O_2_ in the potential range of 0.20 to −0.10 V.

**Figure 5:**
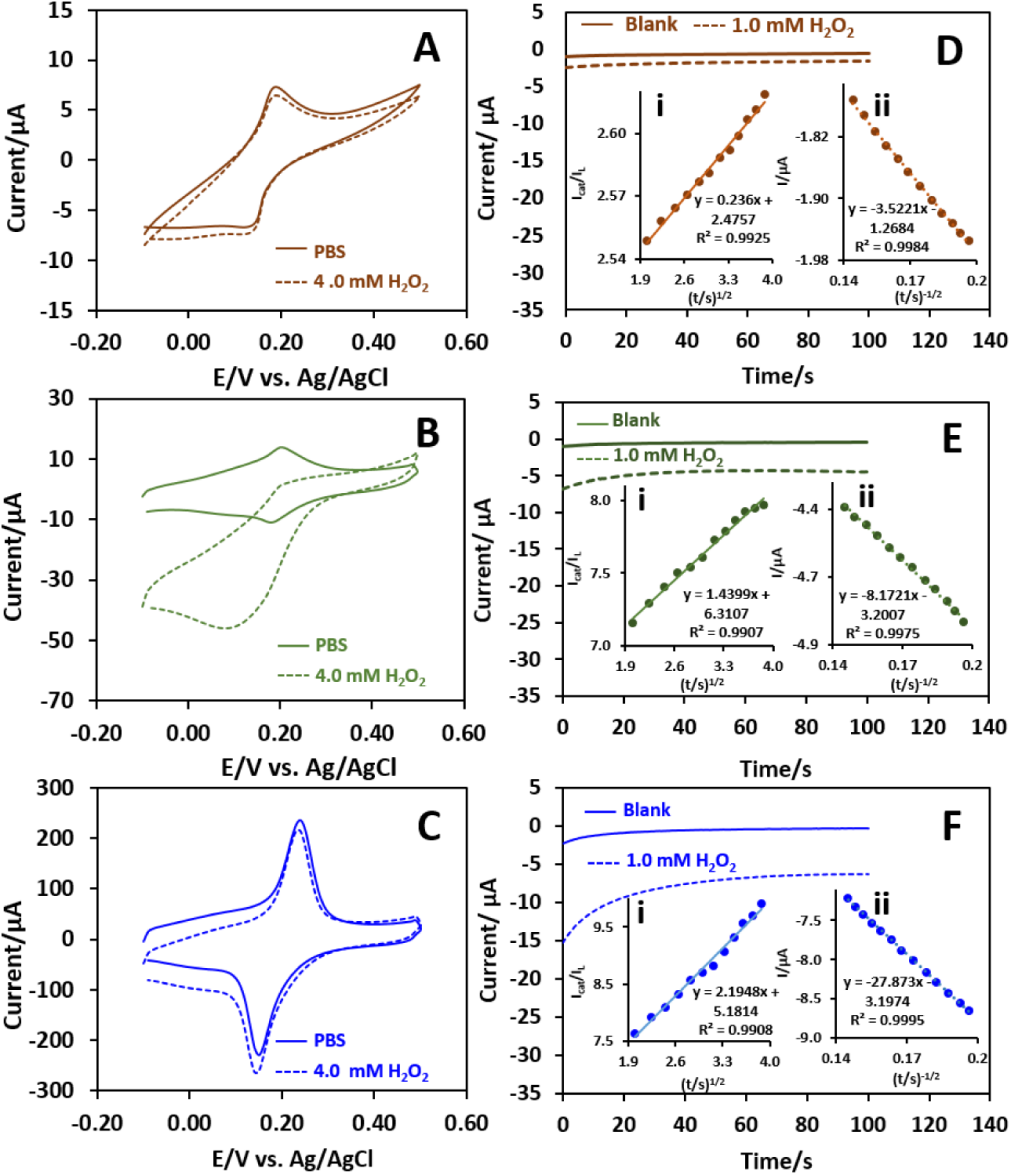
CVs of (A) PB, (B) stepwise PEDOT–PB, and (C) *in situ* PEDOT–PB in PBS in the absence (solid line) and the presence (dotted line) of 4 mM H_2_O_2_ at a scan rate of 0.05 V s^–1^. Chronoamperograms of (D) PB, (E) stepwise PEDOT–PB, and (F) *in situ* PEDOT–PB with (dotted line) and without (solid line) 1 mM H_2_O_2_ at 0.00 V (vs. Ag/AgCl). The insets (i) and (ii) are the plots of *I*_cat_/*I*_L_ vs *t* ^1/2^ and *I* vs *t* ^–1/2^, respectively.

Chronoamperometry was used to determine the catalytic rate constant (*k*_cat_) of H_2_O_2_ reduction on the unary PB, stepwise PEDOT–PB, and *in situ* PEDOT–PB modified electrodes. Figure 5D–F shows the chronoamperograms of different electrodes in PBS with (dotted line) and without (solid line) 1 mM H_2_O_2_ at a potential of 0.00 V vs. Ag/AgCl. The current increased in the negative direction due to the electrochemical catalytic reduction of H_2_O_2_ at all of the electrodes. The *k*_cat_ value can be calculated from the slope of *I*_cat_/*I*_L_ vs. *t* ^1/2^ plots (inset i of Figure 5D–F) based on the Galus equation (eq 3):^45^

3 $$I_{\mathrm{Cat}}/I_{L}=\pi^{1/2}{\left(k_{\mathrm{cat}}C_{b}t\right)}^{1/2}$$

where *I*_Cat_ is the catalytic current of H_2_O_2_, *I*_L_ is the limiting current in the absence of H_2_O_2_, C is the bulk concentration, and *t* is the time. The *k*_cat_ values were found to be 133, 812, and 1238 M^–1^ s^–1^ for the unary PB, stepwise PEDOT–PB, and *in si*tu PEDOT–PB, respectively.

In addition, the diffusion coefficient (*D*) of H_2_O_2_ at the unary PB, stepwise PEDOT–PB, and *in situ* PEDOT–PB was obtained from the slope of the plot of *I* versus *t* ^–1/2^ (inset ii of Figure 5D–F) according to the Cottrell equation (eq 4):^42^

4 $$I={\mathrm{nFACD}}^{1/2}/\pi^{1/2}t^{1/2}$$

where *n* is the number of electrons transferred, *A* is the electrode surface area (cm^2^), *C* is the bulk concentration (mM), *t* is time (s), and *D* is the diffusion coefficient (cm^2^ s^–1^). The *D* values of H_2_O_2_ at the unary PB, stepwise PEDOT–PB, and *in situ* PEDOT–PB were calculated to be 8.5 × 10^–7^, 4.6 × 10^–6^, and 5.3 × 10^–5^ cm^2^ s^–1^, respectively. These results indicate the superior catalytic activity and mass transport properties of the *in situ* PEDOT–PB electrode for the reduction of H_2_O_2_ compared to the unary PB and stepwise PEDOT–PB electrodes, which is facilitated by the heterostructure of the conductive PEDOT matrix for relatively fast H_2_O_2_ diffusion and well-embedded PB nanoparticles for the electrocatalytic reaction.

In practical applications, especially for H_2_O_2_ reduction in sensors and biosensors, biofuel cells, and batteries, the stability of PB is usually hindered by dissolution via the reaction of Fe with HO^–^ that originates from H_2_O_2_ reduction, resulting in the progressive loss of catalytic activity. The electrocatalytic stability of unary PB, stepwise PEDOT–PB, and *in situ* PEDOT–PB to 1 mM H_2_O_2_ was investigated by chronoamperometry in a batch and flow injection system. For the batch system, Figure 6A–C displays the chronoamperograms of unary PB, stepwise PEDOT–PB, and *in situ* PEDOT–PB electrodes after the addition of 1 mM H_2_O_2_ with continuous stirring at 250 r.p.m. over a period of 3000 s at a potential of 0.00 V (vs. Ag/AgCl). The unary PB electrode showed a current response of 8.92 μA toward the injection of 1 mM H_2_O_2_, while the response decreased dramatically, with only 4.37% retention of its initial value. This obvious decrease in the catalytic current is caused by the direct exposure of PB nanoparticles to the intermediate product (HO^–^) of H_2_O_2_ reduction. As a comparison, the current response to 1 mM H_2_O_2_ at the stepwise PEDOT–PB electrode is 13.5 μA. The curve maintained a relatively stable response in the first 1000 s, while it gradually decreased to 25.7% of its initial value at 3000 s. The current response of the *in situ* PEDOT–PB electrode is 21.8 μA, which is 2.4 and 1.6 times higher than that of the unary PB and stepwise PEDOT–PB electrodes, respectively, and it possesses the highest stability of the electrocatalytic response, retaining 80.7% of the initial current after 3000 s. This demonstrates the excellent electrocatalytic stability of *in situ* PEDOT–PB.

**Figure 6:**
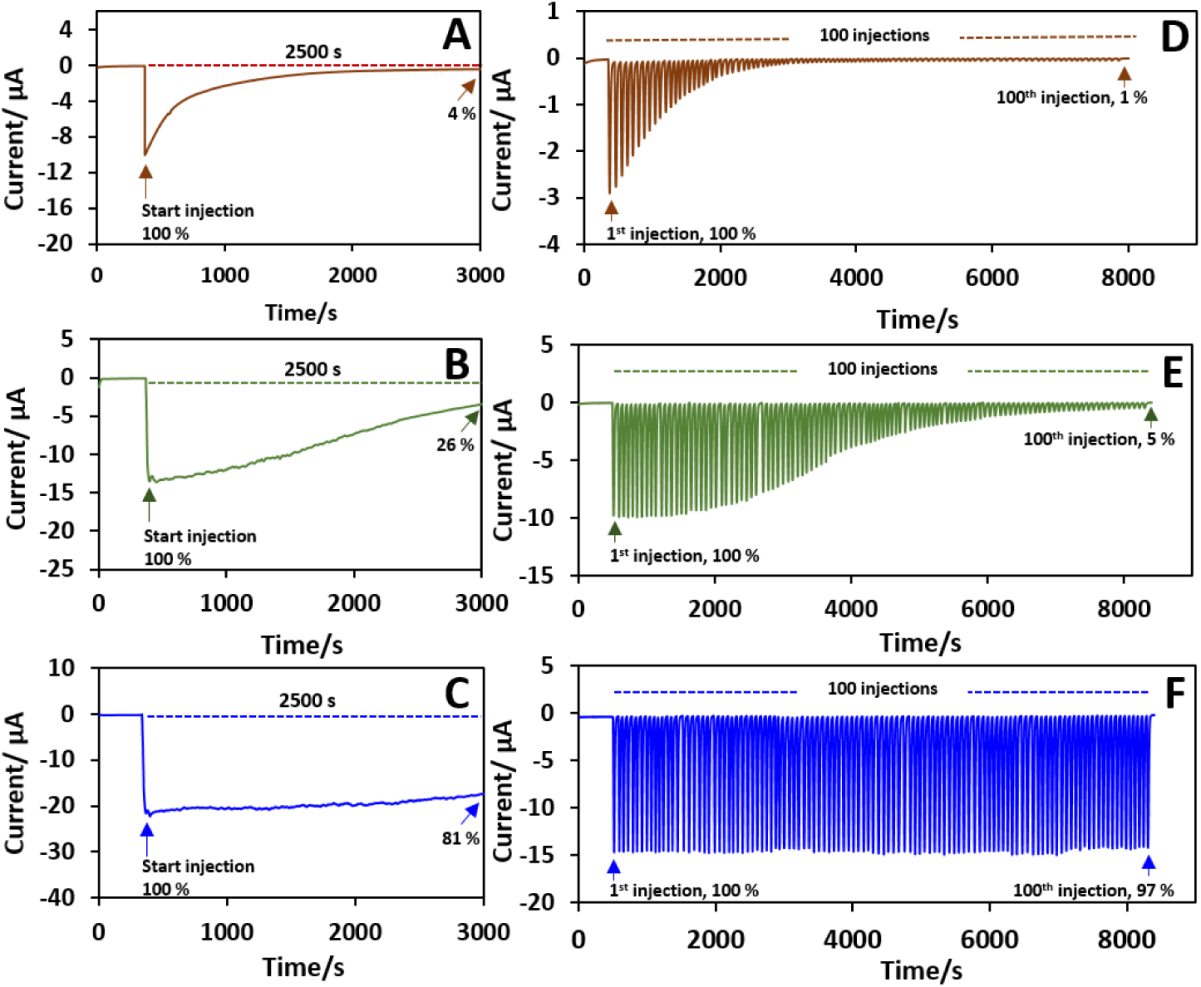
Electrocatalytic stability in batch and flow injection systems of (A, D) PB, (B, E) stepwise PEDOT–PB, and (C, F) *in situ* PEDOT–PB to 1 mM H_2_O_2_ in 0.10 M PBS.

The operational stability of H_2_O_2_ reduction at the unary PB, stepwise PEDOT–PB, and *in situ* PEDOT–PB electrodes was further investigated via flow injection analysis (FIA) by switching on/off the catalytic reaction. The FIA was performed with repeated injections of 1 mM H_2_O_2_ for 100 injection cycles using PBS as a carrier solution under a flow rate of 1 mL min^–1^ and an injection volume of 300 μL at a potential of 0.00 V. The recorded flow injection chronoamperograms are shown in Figure 6D–E. After 100 injection cycles, the electrocatalytic current response of the unary PB, stepwise PEDOT–PB, and *in situ* PEDOT–PB remained at 1.31%, 5.05%, and 96.6% of the initial response, respectively. These results suggest that *in situ* PEDOT–PB displays excellent switchability and electrocatalytic stability for H_2_O_2_ reduction. In addition, the standard deviation of the response to the 100 injections of 1 mM H_2_O_2_ at the *in situ* PEDOT–PB was 2.46%, indicating good reusability of the platform in real applications. Furthermore, the electrocatalytic performance of *in situ* PEDOT–PB toward H_2_O_2_ reduction was compared to another two-step PEDOT–PB_chemical_, i.e., chemically synthesized PB embedded in an electropolymerized PEDOT matrix, showing better catalytic H_2_O_2_ reduction from the aspects of *k*_cat_ value, *D* value, and catalytic stability for the *in situ* PEDOT–PB (details in Figure S10). This confirms that the electrocatalytic stability of PB can be improved by the PEDOT matrix via the progressive electrochemical synthesis of PEDOT–PB. The improved stability of the in situ PEDOT–PB composite may be attributed to its effective inhibition of hydroxyl radicals (HO•). In this system, the PB component acts as a redox mediator capable of scavenging HO• via electron transfer. Specifically, the Fe­(II)/Fe­(III) redox couple in PB can reduce hydroxyl radicals generated during the electrochemical reduction of H_2_O_2_.^7^ In addition, the PEDOT matrix contributes by serving as a physical barrier, limiting radical penetration and mitigating PB degradation. This dual mechanism of chemical scavenging by PB and structural protection by PEDOT collectively enhances the oxidative stability and durability of the composite film.

The sensing performance of the *in situ* PEDOT–PB transducer for detecting H_2_O_2_ was systematically examined. Experiments were carried out in 0.10 M PBS (pH 6.6) containing 0.10 M KCl, where different concentrations of H_2_O_2_ were gradually introduced. Under constant stirring and an applied potential of 0.00 V vs Ag/AgCl, the sensor exhibited a quick and pronounced increase in catalytic current upon each addition of H_2_O_2_ (Figure S12A), confirming its high electrocatalytic activity. A linear calibration curve was obtained between the H_2_O_2_ concentration and its corresponding current response across a wide range of 1.0 μM to 10.0 mM (Figure S12B). The transducer displayed a high sensitivity of 199.98 μA mM^–1^ cm^–2^, and the limit of detection (LOD) was calculated to be 0.33 μM, based on the formula LOD = 3 × standard deviation of the blank/slope. Overall, the *in situ* PEDOT–PB sensor offers excellent sensing performance, including low detection potential, high sensitivity, broad linear range, and low LOD. Moreover, to evaluate the H_2_O_2_ sensing performance in real biological samples, the *in situ* PEDOT–PB transducer was employed to detect H_2_O_2_ in 10-fold diluted human serum, both in the absence and presence of 5, 10, 20, and 40 μM H_2_O_2_ standard solutions (Figure S12C). The endogenous H_2_O_2_ concentration in the serum was successfully determined. Subsequently, known concentrations of H_2_O_2_ (5, 10, 20, and 40 μM) were spiked into the serum samples, and the corresponding current responses were recorded. The calculated recovery values were 104.4%, 98.0%, 99.7%, and 99.9%, respectively, confirming the high accuracy and practical applicability of the *in situ* PEDOT–PB transducer for H_2_O_2_ detection in complex biological matrices.

## 4 Conclusions

In this presented work, we demonstrated a facile one-step preparation of heterostructured PEDOT–PB via an innovative progressive electrochemical deposition approach by modulating the *in situ* electrochemical synthesis of PB and the electropolymerization of PEDOT simultaneously. The *in situ* PEDOT–PB interface exhibited good electrochemical performance and enhanced electrode kinetics, characterized by CV, EIS, and chronoamperometry, compared with unary PB and stepwise PEDOT–PB. More importantly, the heterostructured *in situ* PEDOT–PB delivered excellent electrocatalytic stability (static and flow injection analysis), retaining 80.7% and 96.6% of its initial catalytic performance. In addition, the preparation procedure, performance, and stability of the *in situ* PEDOT–PB were compared with other reported conductive polymer–PB composites in the literature, as summarized in Table S6, thereby demonstrating the advantages of the proposed approach. Our development provides a new solution to tackle the challenges of the facile synthesis of conductive-PB composites and the instability of PB, which is one of the most important inorganic catalysts, for the future development of advanced electrochemical devices such as sensors and biosensors, active electrodes for electrocatalysis, capacitors, and fuel cells.

## Supplementary Material



## Notes

Details of materials and characterization instruments; preparation of comparison samples; influence of scan rate and deposition cycles on *in situ* PEDOT–PB composite; XPS analysis of PEDOT, PB, and *in situ*PEDOT–PB; macroscopic appearance of electrodeposited films; EDS spectra and elemental mapping; additional figures showing morphological changes, EIS Bode plots, cycling stability, chronoamperometric analysis, and calibration plots; and tables summarizing electrochemical parameters, elemental composition, interface properties, EIS data, and performance comparison with literature-reported PB-based composites for H_2_O_2_ sensing (PDF)
