# Supplementary material for: Advanced Heterostructured PEDOT–PB Transducer Interface via One-Step Progressive Electrochemical Deposition for Stable and High-Performance Hydrogen Peroxide Electrocatalysis
Source: ACS Appl Mater Interfaces. 2025 Jul 30;17(32):46115–25. doi: 10.1021/acsami.5c07083 (PMC12356530; doi:10.1021/acsami.5c07083)
Supplement: Supplementary file 1 [file am5c07083_si_001.pdf]

Supporting Information

Advanced heterostructured PEDOT-PB transducer interface via one-step progressive electrochemical deposition for stable and high-performance hydrogen peroxide electrocatalysis

Kiattisak Promsuwan [a,c,d,e], Lingyin Meng [a**], Panote Thavarungkul [d,f], Proespichaya Kanatharana [d,f], Warakorn Limbut [c,d,e], and Wing Cheung Mak [a,b*]

[a] Division of Sensor and Actuator Systems, Department of Physics, Chemistry and Biology, Linköping University, SE-581 83 Linköping, Sweden

[b] Department of Biomedical Engineering, The Chinese University of Hong Kong, Hong Kong SAR, China

[c]Forensic Science Innovation and Service Center, Prince of Songkla University, Hat Yai, Songkhla, 90110, Thailand

[d] Center of Excellence for Trace Analysis and Biosensor, Prince of Songkla University, Hat Yai, Songkhla 90112, Thailand

[e] Division of Health and Applied Sciences, Faculty of Science, Prince of Songkla University, Hat Yai, Songkhla, 90110, Thailand

[f] Division of Physical Science, Faculty of Science, Prince of Songkla University, Hat Yai, Songkhla, 90110, Thailand

* Corresponding author: Email: wing.cheung.mak@cuhk.edu.hk (Wing Cheung Mak).

** Corresponding author: Email lingyin.meng@liu.se (Lingyin Meng).

Materials

3,4-ethylenedioxythiophene (EDOT) monomer (97%), ferrous chloride ($FeCl_2$), ferric chloride ($FeCl_3$) and potassium ferrocyanide ($K_4[Fe(CN)_6]$) were purchased from Sigma Aldrich. Potassium chloride (KCl), and hydrochloride acid (HCl) were purchased from Merck (Germany). 0.1 M phosphate buffer solution (PBS, pH 6.6) containing 0.1 M KCl was prepared by mixing K_2HPO_4 and KH_2PO_4 stocking solution. All chemicals were of analytical grade and used without any further treatment. Deionized water (DI) with a resistivity not lower than 18.2 MΩ·cm obtained from a purification system (Millipore systems, USA) was used thoroughly.

Instruments for characterizations

Scanning electron microscopy (SEM, LEO 155 Gemini, Zeiss, Germany) was used to record the images of the surface morphologies of PB, PEDOT, and PEDOT-PB interfaces. Energy-dispersive X-ray spectroscope (EDS, Oxford Instruments) was employed to determine the chemical compositions. Fourier transform infrared (FTIR) spectrometric measurements were performed by VERTEX (Bruker) equipped with an attenuated total reflection (ATR) measuring cell for investigating the characteristic band.

Preparation of comparison samples

PEDOT: PEDOT film was prepared via the electropolymerization of EDOT monomers (10 mM) in 10 mM HCl solution in the range from -0.50 to 1.20 V for 10 cycles by cyclic voltammetry, at a scan rate of 0.05 V s^{-1}.

PB: PB was electrochemically deposited on the electrode surface by CV in the mixture of $K_4[Fe(CN)_6]$ (5 mM) and $FeCl_2$ (5 mM) in 10 mM HCl, in the range from -0.50 to 1.20 V for 10 cycles at a scan rate of 0.05 V s^{-1}.

Step-wise PEDOT-PB: The electrodeposited solution was prepared by dispersing 10.7 μL EDOT monomer in 10 mL of HCl (10 mM) under sonication for 30 min. After that, 5 mM $K_4[Fe(CN)_6]$ was added to the EDOT solution. Then the cleaned GCE was immersed in the electrodeposited solution and electrodeposited using cyclic voltammetry by potential scanning in the range from -0.50 to 1.20 V for 10 cycles. After that, the electrodeposited electrode was

rinsed and sonicated in deionized water and immersed in 10 mM HCl containing 5 mM $FeCl_2$ with potential scanning in the range from -0.50 to 1.20 V for 10 cycles by cyclic voltammetry. The scan rate was 0.05 V s^{-1}. Finally, step-wise PEDOT-PB/GCE was rinsed with DI water and kept in a dark place at room temperature until used.

Step-wise PEDOT-PB$_{chemical}$: The step-wise PEDOT-PB$_{chemical}$ was prepared via a two-step (wet chemistry and electrochemical method) as follows. Initially, PB nanoparticles were prepared via wet chemical synthesis by quickly mixing 5 mL each of $K_4[Fe(CN)_6]$ (10 mM) and $FeCl_3$ (10 mM) in 10 mM HCl solution under sonication for 30 minutes. Then PB product was washed with acetone by centrifuging at 9000 rpm 2 times and dried at 70 ºC for 2 hours. 10 mM EDOT monomer solution was prepared by dispersing 10.7 µL of EDOT in 10.0 mL of HCl (10 mM) by sonication. The dried PB was dispersed into the EDOT solution to obtain the electrodeposited solution. Then the cleaned GCE was immersed in the electrodeposited solution, and the electrodeposition was carried out by cyclic voltammetry in the range from -0.50 to 1.20 V for 10 cycles at a scan rate was 0.05 V s^{-1}. Finally, step-wise PEDOT-PB$_{chemical}$ /GCE was cleaned with DI water and kept in a dark place at room temperature until further used.

Influence of scan rate and deposition cycles on *in-situ* PEDOT–PB Composite

The cyclic voltammetry (CV) data reveal that lower scan rates tend to produce higher PB deposition, resulting in thicker films with increased charge storage (Q) and capacitance (C). However, these conditions are also associated with larger ΔE values and prolonged deposition times, which are less favorable for sensing applications due to decreased reversibility and potential film instability. In contrast, higher scan rates result in smaller CV areas, shorter deposition times, thinner films with lower PB content, and reduced electrochemical activity (ΔE, Q, and C) (**Figure S1A and Table S1**). Similarly, increasing the number of deposition cycles enhances film thickness and PB loading. However, excessive cycling (e.g., 20 cycles) can lead to providing high ΔE, Q, and C values. On the other hand, using too low deposition cycles (e.g., 5 cycles) yields smaller CV areas with lower ΔE, Q, and C, and produces thin films with insufficient PB content despite shorter deposition times (**Figure S1B and Table S2**). Therefore, achieving an optimal balance between scan rate and cycle number is crucial for fabricating reproducible, stable, and high-performance composite films. Based on these

findings, a scan rate of 0.05 V s^{-1} and 10 deposition cycles were selected as the optimal conditions for this study.

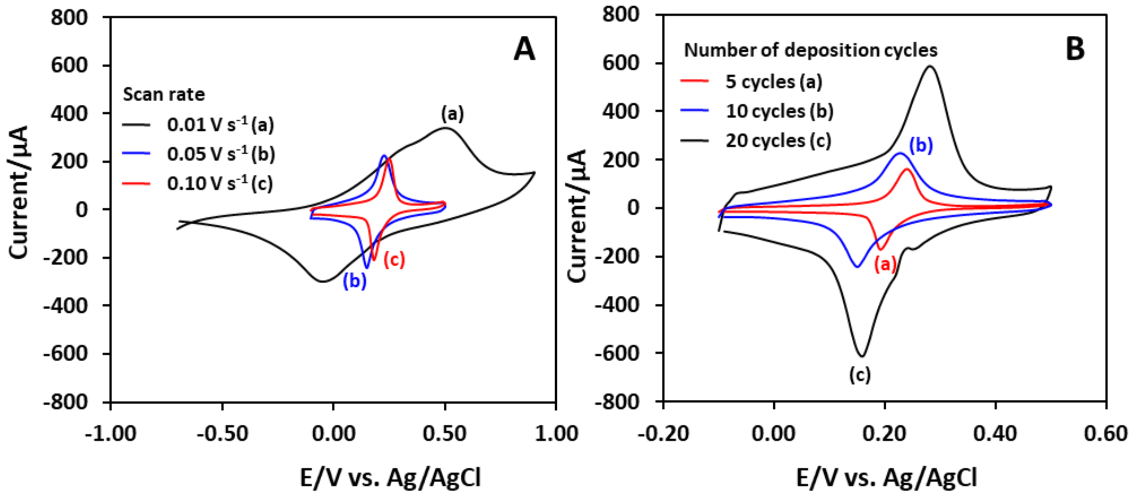

Figure S1. CVs of PEDOT-PB composite films prepared at (A) different scan rates (0.01, 0.05, and 0.10 V s^{-1}) for 10 deposition cycles. (B) The number of deposition cycles (5, 10, and 20 cycles) at a fixed scan rate of 0.05 V s^{-1}.

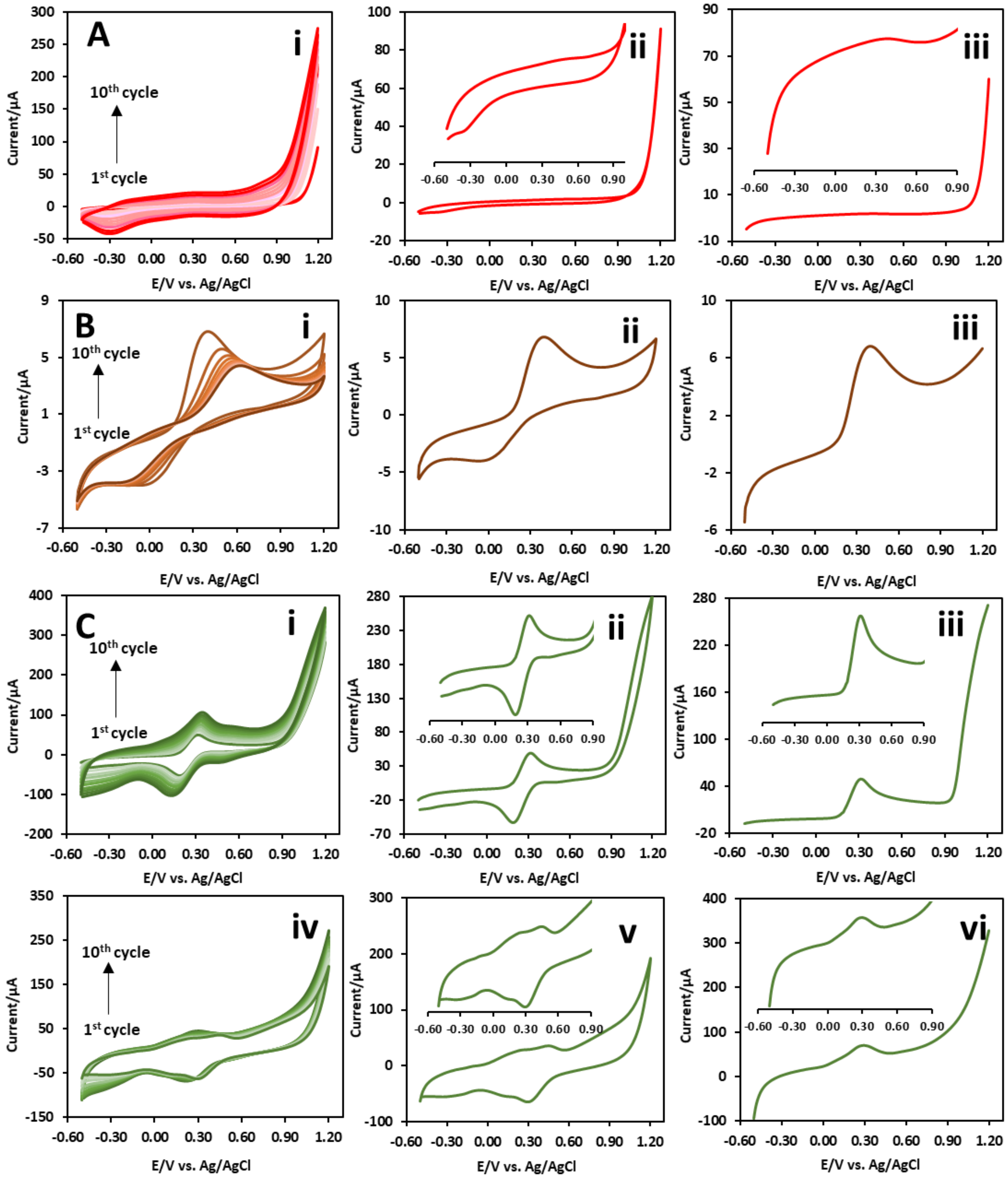

Figure S2. CVs and LSVs of electrodeposition of the (A) PEDOT, (B) PB, and (C) step-wise PEDOT-PB; (i and iv) CVs 10 cycles, (ii and v) CVs at 1st cycle, and (ii and vi) LSVs a first scan.

XPS Analysis of PEDOT, PB, and the *in-situ* PEDOT–PB composite

X-ray photoelectron spectroscopy (XPS) was employed to analyze the surface chemical composition of PEDOT, PB, and the in-situ PEDOT-PB composite (**Figure S3**). For PEDOT, (**Figure S3A**) signals corresponding to O, C, and S were clearly observed. The high-resolution O1s spectrum showed peaks at 532.1 eV and 533.2 eV, attributed to C–O–C in the PEDOT backbone and C–O or residual –OH groups, respectively [1]. The C 1s spectrum revealed peaks at 283.9 eV (C–C/C–H), 285.3 eV (C=C–O or C–S), 286.6 eV (C–O–C), and 287.5 eV (C–O), while the S 2p spectrum exhibited peaks at 163.2 eV S ($2p_{1/2}$) and 164.3 eV ($2p_{3/2}$) for thiophene sulfur, along with additional peaks at 165.5 eV and 166.5 eV corresponding to oxidized sulfur species [2-4]. For the PB film (**Figure S3B**), Fe, N, and C were detected. The Fe 2p spectrum showed peaks at 708.6 eV and 721.3 eV (Fe(II) $2p_{3/2}$ and $2p_{1/2}$) and at 711.3 eV and 723.7 eV (Fe(III)), indicating mixed-valence iron in the Fe–C≡N–Fe framework [4, 5]. The N 1s spectrum displayed peaks at 397.2 eV and 398.8 eV, corresponding to Fe(II)–N≡C and Fe(III)–N≡C, and a peak at 401.8 eV attributed to oxidized nitrogen [6]. The C 1s spectrum showed peaks at 288.9 eV (C–O or C=N), 285.8 eV (C≡N), and 284.3 eV (C–C) [7]. In the in-situ PEDOT-PB composite (**Figure S3C**), the survey spectrum confirmed the presence of Fe, O, N, C, and S, indicating successful integration of both components. The Fe 2p spectrum retained characteristic the Fe $2p_{3/2}$ peak at 710.6 eV and Fe $2p_{1/2}$ at 724.0 eV confirm the presence of Fe(III) in the PB lattice, with subtle spectral features suggesting Fe-S interactions, likely due to coordination between Fe centers in PB and sulfur atoms in the PEDOT thiophene rings. The O 1s peak at 531.8 eV was attributed to the ethylenedioxy groups in PEDOT and adsorbed oxygen species. The N 1s and C 1s spectra showed signals consistent with contributions from both PB and PEDOT, while the S 2p doublet at 163.6 and 164.8 eV confirmed thiophene sulfur, with slight shifts further supporting Fe-S coordination [3]. These results confirm the successful formation of a chemically integrated PEDOT-PB composite with enhanced interfacial interaction.

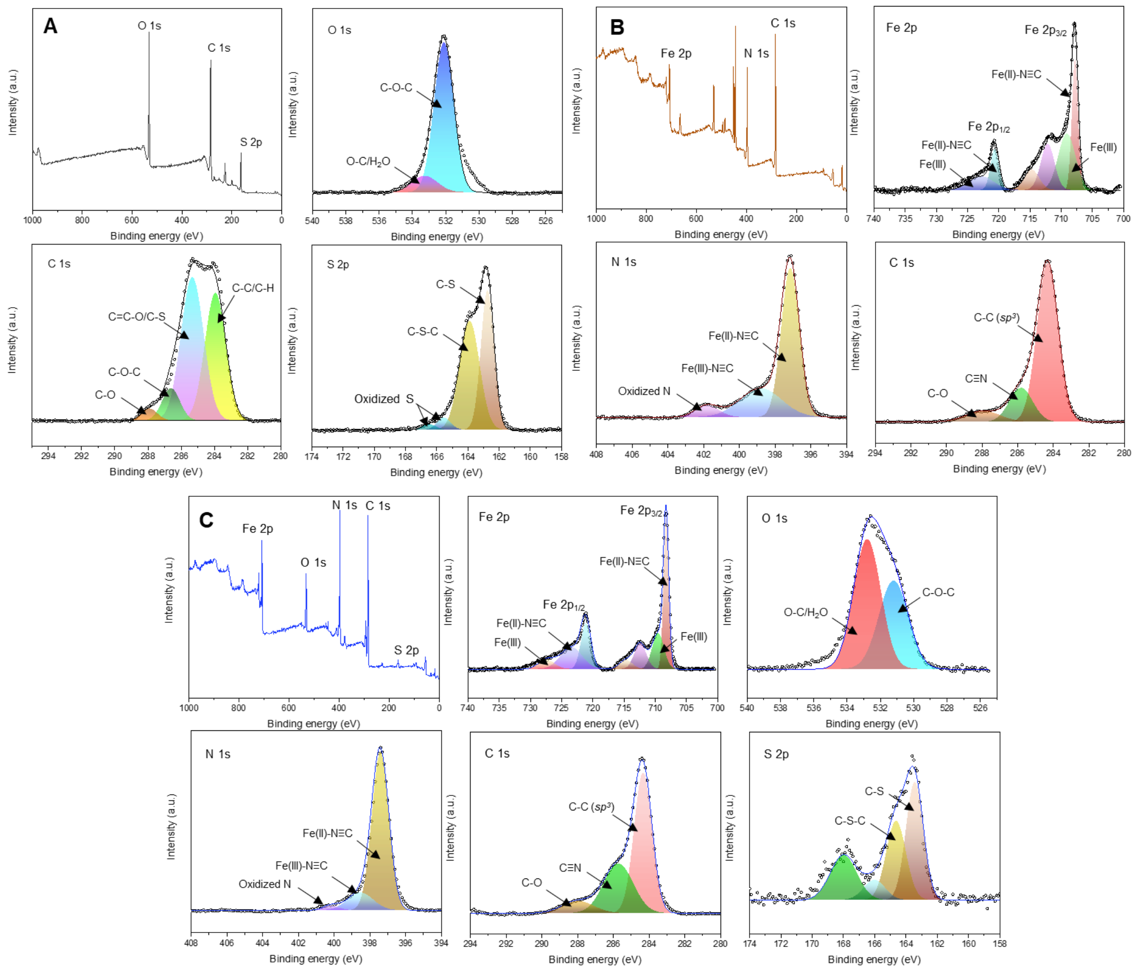

Figure S3. XPS spectrum of the (a) PEDOT, (B) PB, and (C) *in-situ* PEDOT-PB.

The macroscopic appearance of the electrodeposited films

The macroscopic appearance of the films provides visual insight into their surface morphology and phase distribution. The PB film appears light blue and smooth (**Figure S4A**), while the PEDOT film is darker gray-blue tone with a matte texture (**Figure S4B**), suggesting intrinsic microstructural roughness. The PEDOT-PB composite film displays a dark blue to nearly black color with moderate reflectivity (**Figure S4C**), indicating increased density and complexity at the surface. These differences align with SEM and EDS results, which confirm PB-rich domains and particle formation. However, we acknowledge that PEDOT, particularly under varying electrochemical deposition conditions, also contributes to surface texture. The overall roughness of the composite film, therefore, results from the combined effects of both PB crystallite formation and the semi-amorphous structure of the PEDOT matrix.

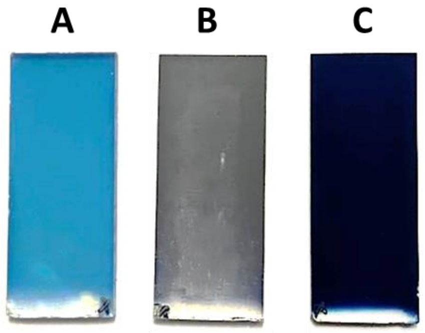

Figure S4. Digital images of electrodeposited films under ambient light (A) PB, (B) PEDOT, and (C) *in-situ* PEDOT-PB film.

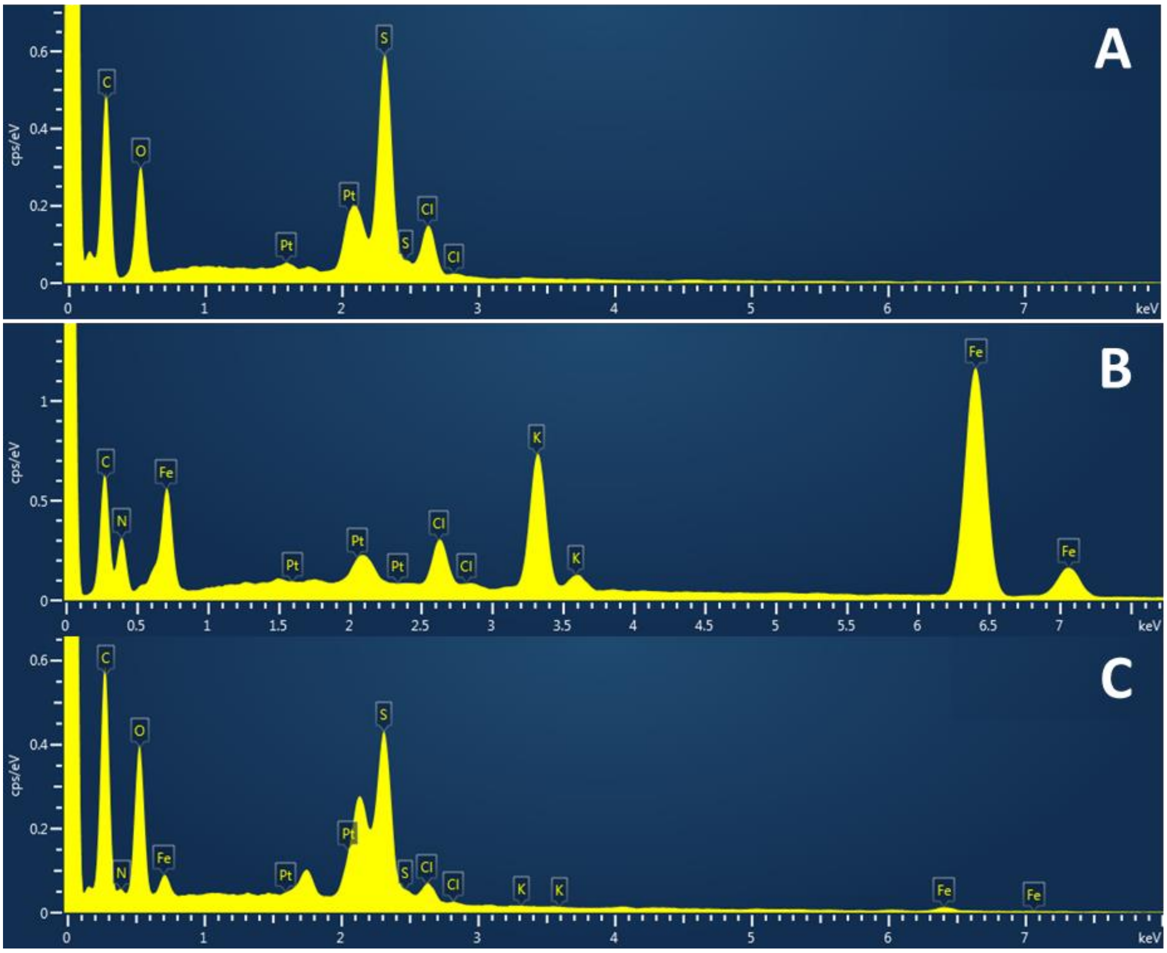

Figure S5. EDS spectra of the (A) PEDOT, (B) PB, and (C) step-wise PEDOT-PB

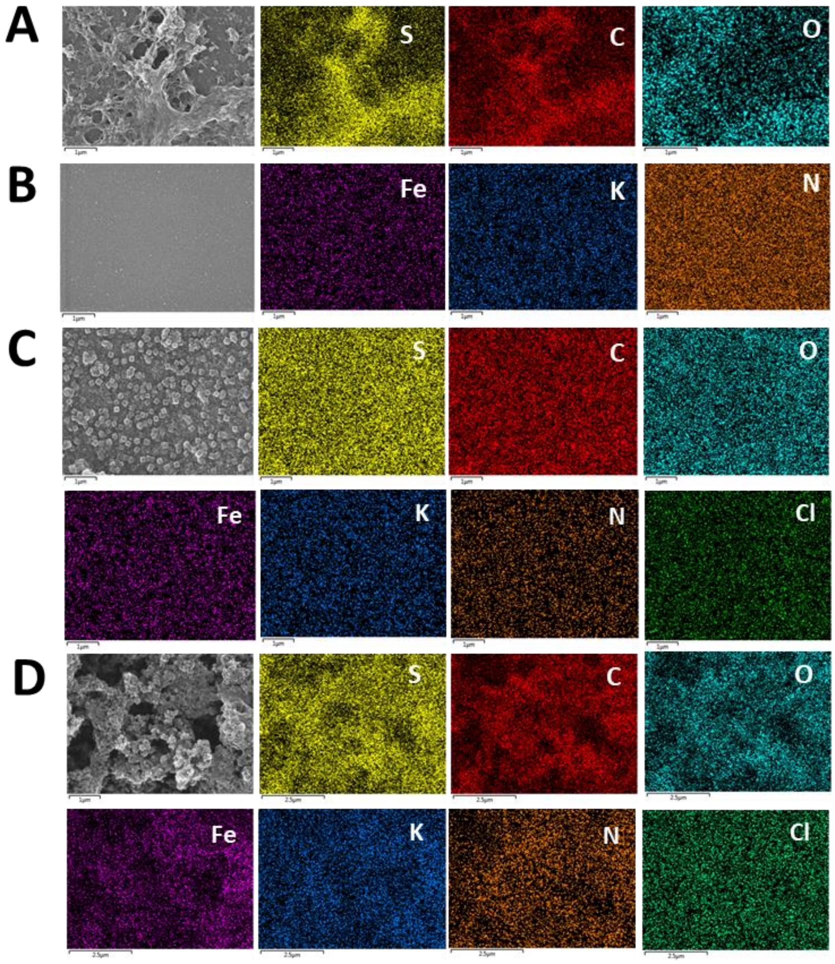

Figure S6. EDS mapping of the (A) PEDOT, (B) PB, (C) step-wise PEDOT-PB, and (D) *in-situ* PEDOT-PB

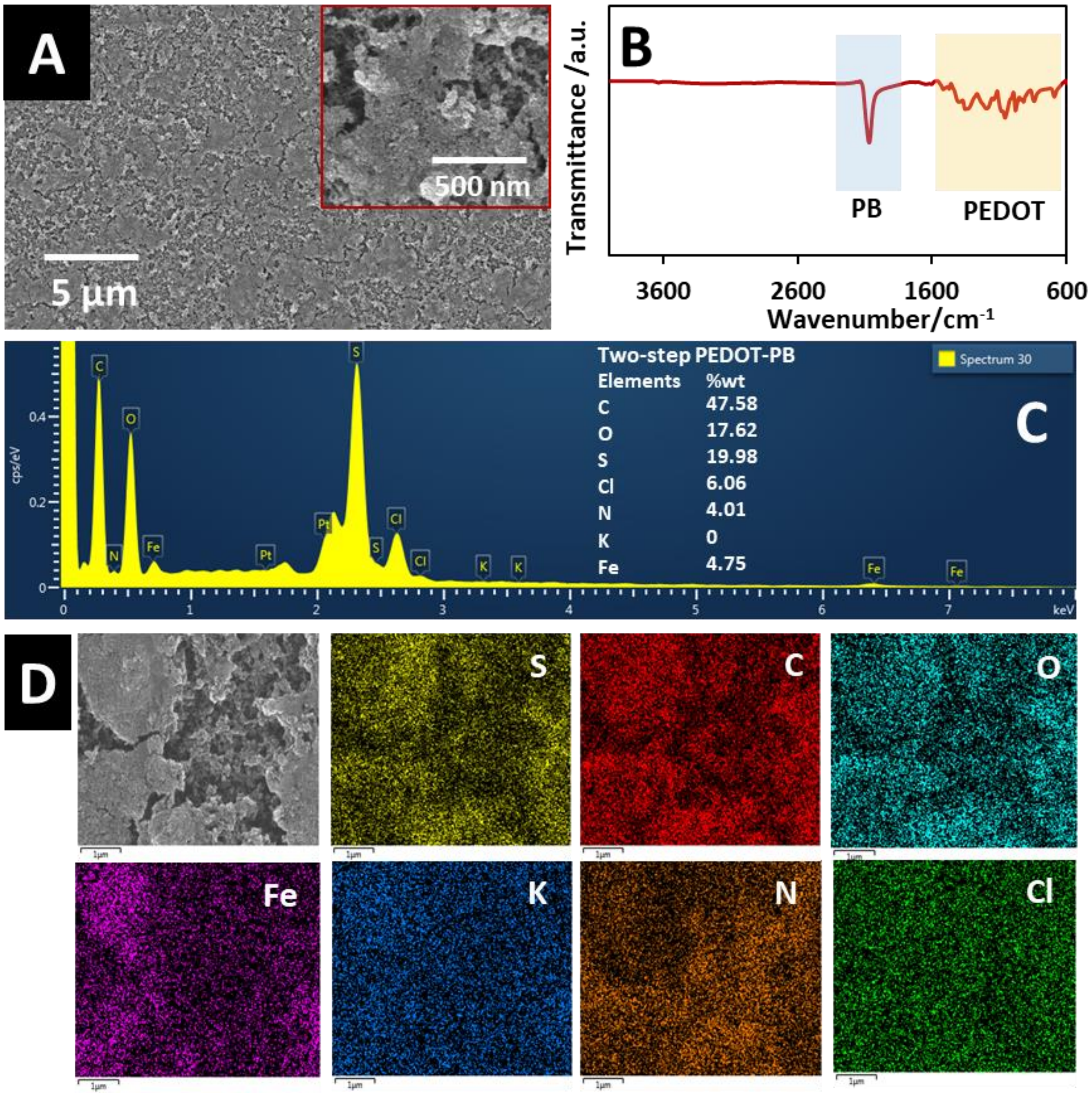

Figure S7. (A) SEM images, (B) FT-IR spectrum, (C) EDS spectra and (D) EDS mapping of the two-step PEDOT-PB (chemical and electrochemical method)

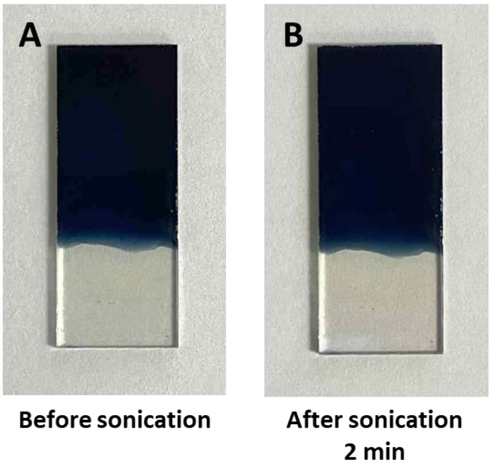

Figure S8. Digital images showing the morphological change before (A) and after (B) sonication treatment.

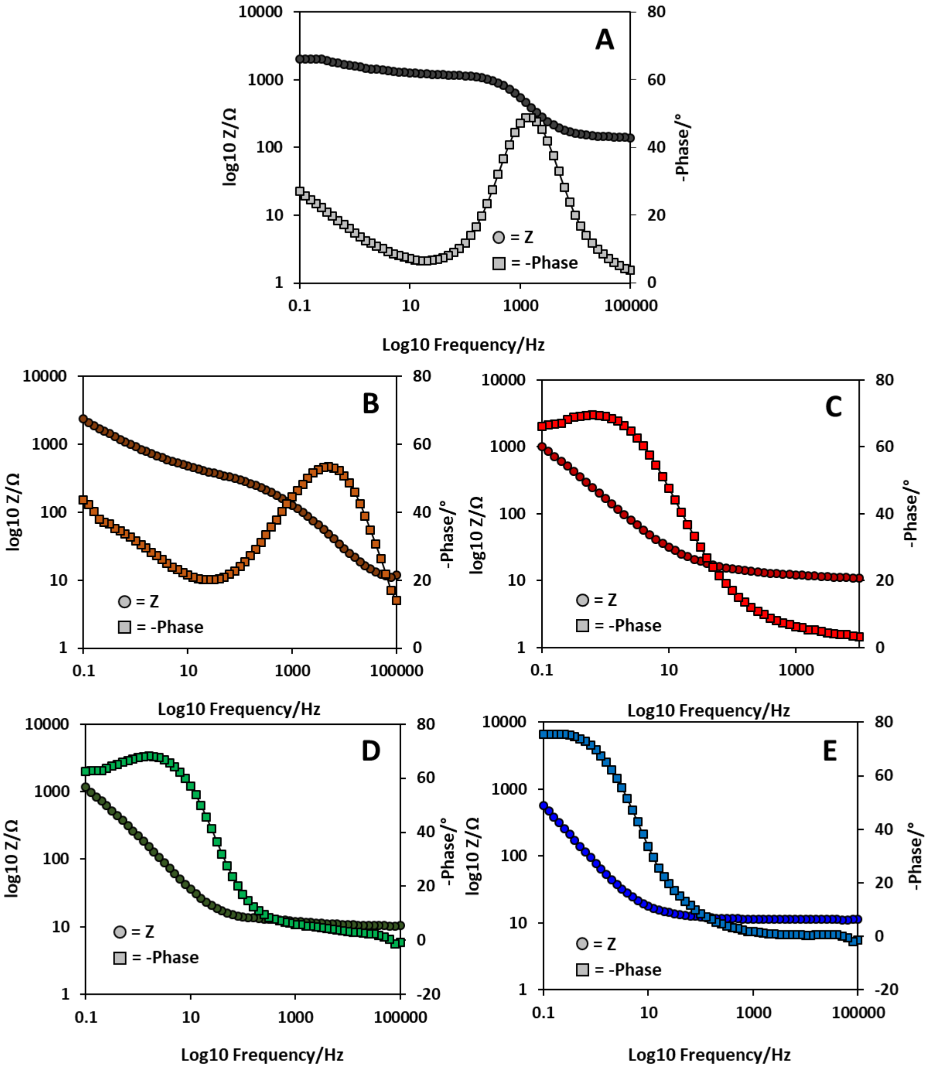

Figure S9. Bode plots for EIS measurement of bare GCE (A), PB (B), PEDOT (C), step-wise PEDOT-PB(D), and *in-situ* PEDOT-PB (E).

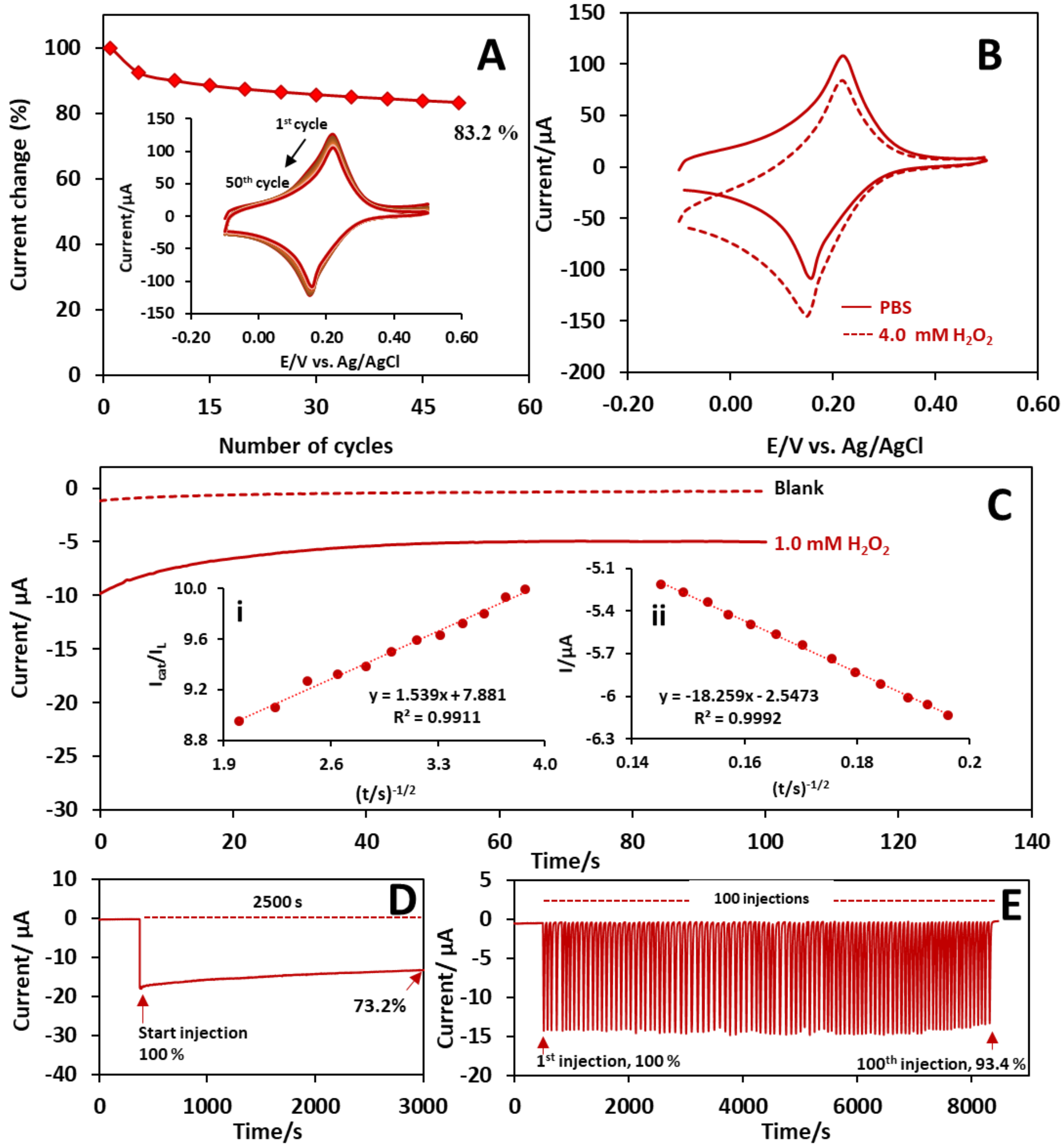

Figure S10. (A) Cycling stability (B) CVs in the absence and the presence of 4.0 mM H_2O_2, (C) Chronoamperogram with the plot of I_{cat}/I_L vs. $t^{1/2}$ (inset (a)) and I vs. $t^{-1/2}$ (inset (b)), Electrocatalytic current stability in the batch (D) and flow injection system (E) of the two-step PEDOT-PB (chemical and electrochemical method)

The electrochemical performance including the cyclic stability of and catalytic performance of the chemically synthesized PB integrated with PEDOT toward H_2O_2 detection was compared with the *in-situ* PEDOT-PB, in which PB was synthesized by bulk chemical method and electrodeposition with PEDOT, respectively **(Section S2).** The result showed that the cyclic stability of the chemical synthesis PB integrated with PEDOT was 83.2% (**Figure S10A)** and the chemically synthesized PB integrated with PEDOT provides good catalytic reduction of H_2O_2 (**Figure S10B**), catalytic rate constant of 868 M^{-1} s^{-1} (**Figure S10C-i**), diffusion coefficient of 2.3 $\times 10^{-5}$ cm^2 s^{-1} (**Figure S10C-ii**), electrocatalytic stability 73.2% (batch) (**Figure S10D**) and 93.4% (flow injection system) (**Figure S10E**). All of these parameters were slightly lower than those obtained from the *in-situ* PEDOT-PB .These comparable results demonstrated that the *in-situ* PEDOT-PB provides a versatile and promising electrocatalyst for electrochemical application.

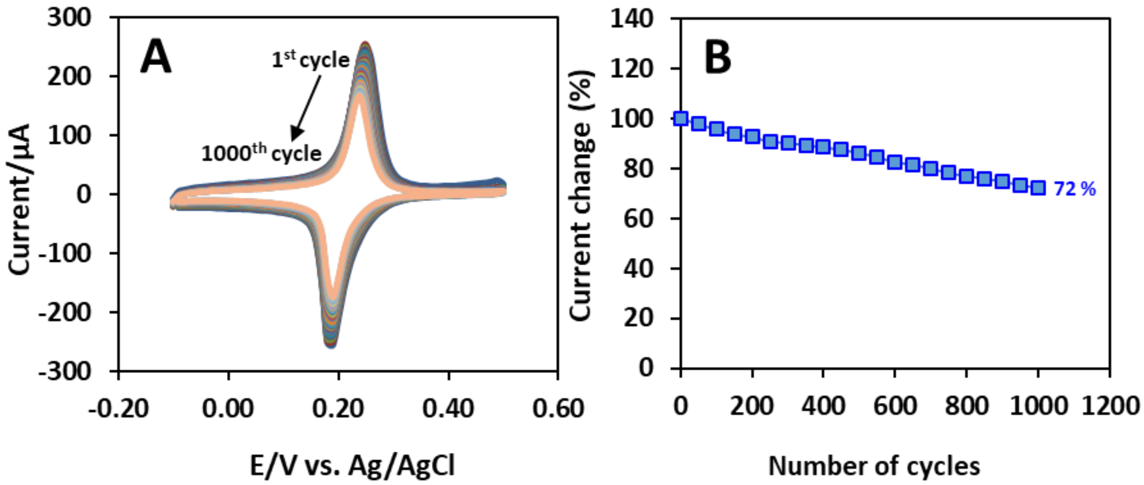

Figure S11. CVs of (A) in-situ PEDOT-PB in PBS at a scan rate of 0.05 V s^{-1}. (B) Anodic peak current changes of in-situ PEDOT-PB for 1000 cycles.

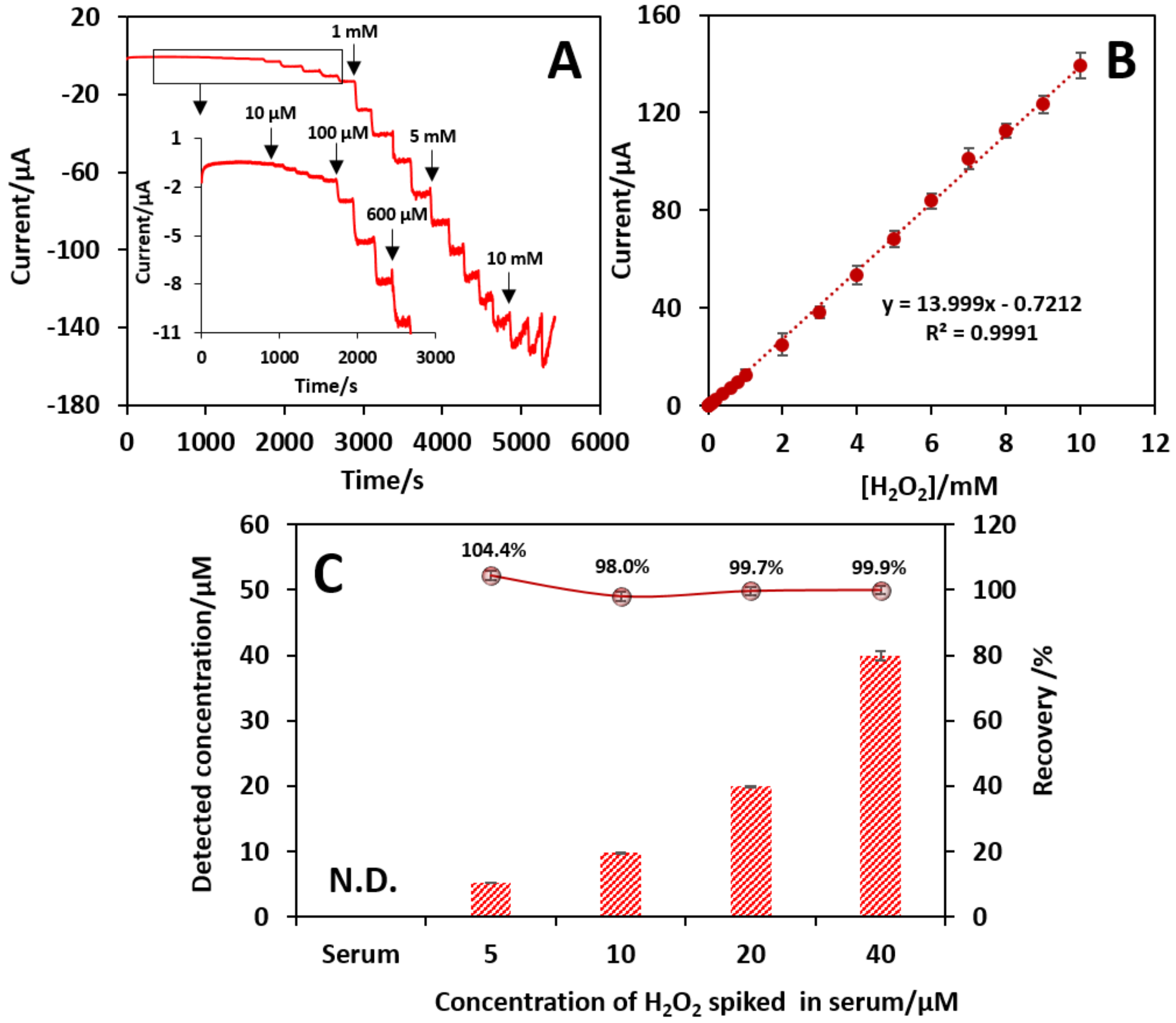

Figure S12. (A) Chronoamperogram of *in-situ* PEDOT-PB toward successive addition of H_2O_2 over the range of 0.001 to 10 mM in 0.10 M PBS (pH 6.6) at 0.00 V, (B) the corresponding calibration plot, and (C) practical determination of H_2O_2 levels in a 10-fold diluted human serum sample spiked with 15, 10, 20, and 40 μM of H_2O_2, along with the corresponding recovery values.

Table S1. Summary of electrochemical parameters obtained from PEDOT–PB films deposited at different scan rates.

Scan rate ($V s^{-1}$)	ΔE (mV)	Q (μC)	Γ_{PB} ($nmol\ cm^{-2}$)	C_{areal} ($mF\ cm^{-2}$)	Thickness (μm)	Deposition time (min)
0.01	539	3730	94.76	1776.1	0.713	56.45
0.05	70	1340	26.43	638.1	0.256	11.22
0.10	70	838	11.62	399	0.16	5.42

Table S2. Summary of electrochemical data for PEDOT–PB composites deposited using varying numbers of cycles at a scan rate of 0.05 $V s^{-1}$.

Number of cycles	ΔE (mV)	Q (μC)	Γ_{PB} ($nmol\ cm^{-2}$)	C_{areal} ($mF\ cm^{-2}$)	Thickness (μm)	Deposition time (min)
5	59	565	8.95	269	0.108	5.42
10	70	1340	26.43	638.1	0.256	11.22
20	120	4150	614.44	1976.1	0.794	22.42

Table S3. Percentage composition of elemental atoms on different PEDOT, PB and PEDOT-PB modified electrodes obtained from EDS analysis.

Interfaces	Elemental atom (At%)							Ratio	
	C	O	S	Cl	N	K	Fe	S/C	Fe/C
PEDOT	69.65	14.93	12.02	3.40	0.00	0.00	0.00	0.17	-
PB	43.89	0.00	0.00	0.82	41.07	4.09	10.13	-	0.23
Step-wise PEDOT-PB	62.87	19.38	8.17	1.31	5.60	0.06	2.62	0.13	0.04
In-situ PEDOT-PB	54.34	13.70	7.45	0.75	12.60	4.85	6.31	0.14	0.12

Table S4. Interface properties of different PEDOT and PB-modified electrodes

Electrodes	Q (μC)	Γ ($nmol\ cm^{-2}$)
Bare GCE	0.078	-
PEDOT/GCE	1.99	-
PB/GCE	21.5	1.59
Step-wise PEDOT-PB/GCE	30.1	2.23
In-situ PEDOT-PB/GCE	357	26.43

Table S5. EIS parameters were obtained by fitting the Nyquist curve from the different modified electrode

Electrodes	R_{ct} (Ω)	R_s (Ω)	W(mΩ$s^{-1/2}$)	CPE (mF)
Bare GCE	297.06	143.86	1.24	0.010
PEDOT/GCE	17.01	147.32	1.93	0.752
PB/GCE	71.03	140.81	1.54	0.425
Step-wise PEDOT-PB/GCE	4.72	147.30	1.33	0.912
In-situ PEDOT-PB/GCE	0.57	146.20	1.19	0.749

Table S6. Comparison of the developed in-situ PEDOT–PB composite utilized for H_2O_2 sensing with other polymer-based PB composites reported in the literature.

Polymer-PB-based transducer	Preparation procedure	Controlable Thickness	Linear range (mM)	ΔE (mV) (Smaller value indicates faster electron transfer kinetics)	Sensitivity (μA mM^{-1} cm^{-2})	Stability	Ref.
PANI-PB	Multi-step	No	0-0.50	195	N.R.	N.R.	8
PAA -PB	Multi-step	Yes	N.R.	180	N.R.	N.R.	9
Step-wise PB-PEDOT	Multi-step	No	0.010-1.76	260	344.1	N.R.	10
Step-wise PB-PEDOT	Multi-step	Yes	0.5-839	N.R.	N.R.	50 cycles /83%	11
in-situ PEDOT–PB	One-step	Yes	0.001-10	70	199.9	50 cycles /97%	This work

N.R. = Report

References

(1) Lisowska-Oleksiak, A.; Nowak, A. P.; Wilamowska, M.; Sikora, M.; Szczerba, W.; Kapusta, C. Ex situ XANES, XPS and Raman studies of poly(3,4-ethylenedioxythiophene) modified by iron hexacyanoferrate. Synth. Met. 2010, 160 (11), 1234-1240.

(2) Spanninga, S. A.; Martin, D. C.; Chen, Z. X-ray Photoelectron Spectroscopy Study of Counterion Incorporation in Poly(3,4-ethylenedioxythiophene) (PEDOT) 2: Polyanion Effect, Toluenesulfonate, and Small Anions. J. Phys. Chem. C. 2010, 114 (35), 14992-14997.

(3) Azman, N. H. N.; Lim, H. N.; Mamat, M. S.; Sulaiman, Y. Synergistic Enhancement of Ternary Poly(3,4-ethylenedioxythiophene)/Graphene Oxide/Manganese Oxide Composite as a Symmetrical Electrode for Supercapacitors. Energies 2018, 11 (6), 1510.
(4) Lisowska-Oleksiak, A.; Nowak, A. P.; Jasulaitiene, V. Poly(3,4-ethylenedioxythiophene)-Prussian Blue hybrid material: Evidence of direct chemical interaction between PB and pEDOT. Electrochem. commun. 2006, 8 (1), 107-112.
(5) Lien, S.-Y.; Lin, P.-C.; Chen, W.-R.; Liu, C.-H.; Lee, K.-W.; Wang, N.-F.; Huang, C.-J. The Mechanism of PEDOT: PSS Films with Organic Additives. Crystals 2022, 12 (8), 1109.
(6) Fu, X.; Li, K.; Zhang, C.; Wang, Q.; Xu, G.; Rogachev, A. A.; Yarmolenko, M. A.; Cao, H.; Zhang, H. Homogeneous and Nanogranular Prussian Blue to Enable Long-Term-Stable Electrochromic Devices. ACS Appl. Mater. Interfaces 2024, 16 (14), 17745-17756.
(7) Nawar, A. M.; Alzharani, A. A. Impedance spectroscopy and conduction mechanism analysis of bulk nanostructure Prussian blue pellets. Mater. Chem. Phys. 2023, 306, 128000.
(8) Almanqur, L.; Alharbi, Y. T.; Alderhami, S. A.; Alsulami, A. H.; Saeed, M. A. S.; Almuqrin, A. H.; Nawar, A. M. Optical properties of prussian blue thin films and electrical characteristics of Ag/prussian blue/p-Si/Al photodetectors for low-optical power latch-switching applications. Opt. Mater. 2024, 151, 115162.
(9) Cao, Y.; Shi, H.; Zheng, Y.; Tan, Z.; Xie, Z.; Zhang, C.; Chen, Z. Polyaniline/Prussian blue nanolayer enhanced electrochemical sensing of H2O2 in EBC using an integrated condensation facemask. Sens Actuators B Chem. 2023, 393, 134189.
(10) Gilpin, V.; McCormick, R.; McMath, R.; Smith, R. B.; Papakonstantinou, P.; Davis, J. Evaluating polyanthranilic acid as a polymeric template for the production of Prussian blue nanoclusters. J. Mater. Sci. 2024, 59 (31), 14245-14258.
(11) Meng, L.; Turner, A. P. F.; Mak, W. C. Conducting Polymer-Reinforced Laser-Irradiated Graphene as a Heterostructured 3D Transducer for Flexible Skin Patch Biosensors. ACS Appl. Mater. Interfaces 2021, 13 (45), 54456-54465.
(11) Wang, J.; Wang, Y.; Cui, M.; Xu, S.; Luo, X. Enzymeless voltammetric hydrogen peroxide sensor based on the use of PEDOT doped with Prussian Blue nanoparticles. Mikrochim. Acta. 2017, 184 (2), 483-489.
